# Enzyme‐Responsive Branched Glycopolymer‐Based Nanoassembly for Co‐Delivery of Paclitaxel and Akt Inhibitor toward Synergistic Therapy of Gastric Cancer

**DOI:** 10.1002/advs.202306230

**Published:** 2023-11-12

**Authors:** Xiaohai Song, Hao Cai, Zhaochen Shi, Zhiqian Li, Xiuli Zheng, Kun Yang, Qiyong Gong, Zhongwei Gu, Jiankun Hu, Kui Luo

**Affiliations:** ^1^ Department of General Surgery Gastric Cancer Center Department of Radiology Huaxi MR Research Center (HMRRC) Frontiers Science Center for Disease‐Related Molecular Network Laboratory of Gastric Cancer State Key Laboratory of Biotherapy West China Hospital Sichuan University Chengdu 610041 China; ^2^ Department of Thoracic Surgery and Institute of Thoracic Oncology Frontiers Science Center for Disease‐related Molecular Network West China Hospital of Sichuan University Chengdu 610097 China; ^3^ West China School of Medicine Sichuan University Chengdu 610041 China; ^4^ Functional and Molecular Imaging Key Laboratory of Sichuan Province, West China Hospital, Sichuan University, and Research Unit of Psychoradiology Chinese Academy of Medical Sciences Chengdu 610041 China; ^5^ Department of Radiology West China Xiamen Hospital of Sichuan University Xiamen 361000 China; ^6^ Research Institute for Biomaterials Tech Institute for Advanced Materials College of Materials Science and Engineering NJTech‐BARTY Joint Research Center for Innovative Medical Technology Suqian Advanced Materials Industry Technology Innovation Center Jiangsu Collaborative Innovation Center for Advanced Inorganic Function Composites Nanjing Tech University Nanjing 211816 China

**Keywords:** branched polymers, drug delivery, enzyme‐responsive nanomedicine, gastric cancer, synergistic therapy

## Abstract

Combined chemotherapy and targeted therapy holds immense potential in the management of advanced gastric cancer (GC). GC tissues exhibit an elevated expression level of protein kinase B (AKT), which contributes to disease progression and poor chemotherapeutic responsiveness. Inhibition of AKT expression through an AKT inhibitor, capivasertib (CAP), to enhance cytotoxicity of paclitaxel (PTX) toward GC cells is demonstrated in this study. A cathepsin B‐responsive polymeric nanoparticle prodrug system is employed for co‐delivery of PTX and CAP, resulting in a polymeric nano‐drug BPGP@CAP. The release of PTX and CAP is triggered in an environment with overexpressed cathepsin B upon lysosomal uptake of BPGP@CAP. A synergistic therapeutic effect of PTX and CAP on killing GC cells is confirmed by in vitro and in vivo experiments. Mechanistic investigations suggested that CAP may inhibit AKT expression, leading to suppression of the phosphoinositide 3‐kinase (PI3K)/AKT signaling pathway. Encouragingly, CAP can synergize with PTX to exert potent antitumor effects against GC after they are co‐delivered via a polymeric drug delivery system, and this delivery system helped reduce their toxic side effects, which provides an effective therapeutic strategy for treating GC.

## Graphical abstract

A cathepsin B‐responsive polymeric nanoparticle prodrug system is established for co‐delivery of paclitaxel (PTX) and capivasertib (CAP) to obtain BPGP@CAP, which selectively accumulates in the tumor tissue and releases PTX and CAP in response to overexpressed cathepsin B in lysosomes to exert a synergistic therapeutic effect to eradicate gastric cancer cells.

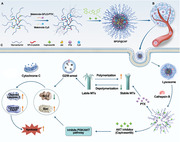

## 1 Introduction

Gastric cancer (GC) is one of the most prevalent malignant tumors globally. Owing to its indistinct early‐stage symptoms, most patients have already progressed into advanced stages at the time of initial diagnosis, resulting in poor overall prognosis.^[^ ^1^ ^]^ Chemotherapy is still the mainstream workforce for advanced GC.^[^ ^2^ ^]^ Paclitaxel (PTX), one of chemotherapeutic drugs in the first‐line regimen for advanced GC, is a cell cycle‐specific antitumor drug that inhibits mitosis and proliferation of tumor cells.^[^ ^3^ ^]^ Despite its excellent antitumor effect against GC, PTX shares the similar issues as other chemotherapeutic agents, such as poor water solubility, lack of targeting specificity, a short retention time, and severe toxic side effects, which have hampered its widespread use in clinical practice.^[^ ^4^ ^]^

The combination of chemotherapy and molecular targeted therapy could synergistically exert antitumor effects, holding an immense prospect of its application in treating GC.^[^ ^5^ ^]^ Currently, the majority of targeted drugs for GC are anti‐human epidermal growth factor receptor 2 or anti‐epidermal growth factor receptor gene therapy drugs, and immunotherapy drugs.^[^ ^6^ ^]^ However, positive response rates of these targeted agents for GC are relatively low, and only a very few patients can benefit from targeted therapy. Significant efforts have been dedicated to the identification of novel targeted drug candidates. The phosphatidylinositol‐3‐kinase (PI3K)/Protein kinase B (AKT) signaling pathway has a crucial regulatory function in cellular processes including cell growth, proliferation, differentiation, apoptosis and glucose transport.^[^ ^7^ ^]^ Activation of this signaling pathway is commonly observed in different types of solid tumors and its activation may be associated with poor prognosis.^[^ ^8^ ^]^ A retrospective study revealed that positive expression of AKT was detected in 81.54% of GC tissues, notably higher than that in 20.8% of normal tissues.^[^ ^9^ ^]^ Abnormal activation of the PI3K/AKT signaling pathway enhances the proliferation, metastasis, and drug resistance of GC cells, while suppressing their apoptosis. PI3K and AKT inhibitors have been demonstrated with promising antitumor effects for GC, thus they have great potential as therapeutic targeted agents.^[^ ^10^ ^]^

It was reported that the combination of AKT inhibitors and chemotherapeutic drugs could synergistically triggers apoptosis and impedes tumor growth.^[^ ^11^ ^]^ Capivasertib (CAP), a highly potent pan‐AKT kinase inhibitor, demonstrates a comparable inhibitory efficacy against three different subtypes of AKT. CAP inhibits the phosphorylation of AKT substrates, thereby blocking the PI3K/AKT signaling pathway. As a result, the growth of cells is inhibited, and apoptosis is promoted.^[^ ^12^ ^]^ Nonetheless, several limitations, such as poor water solubility and low bioavailability, have restricted its widespread application. Encouragingly, combined treatment with PTX and CAP has been shown with an enhancement in the level of PTX‐induced tumor cell apoptosis,^[^ ^13^ ^]^ suggesting that their combination could provide a promising new approach for the treatment of GC. It has been reported that patients could tolerate the combination of AKT inhibitors and chemotherapeutic agents, however, they suffer from serious toxic side effects such as diarrhea, infection, neutropenia, rash, and fatigue, which might be attributed to their suboptimal pharmacokinetics and poor biodistribution in the body.^[^ ^14^ ^]^ Hence, effective means of delivering both AKT inhibitors and chemotherapeutic agents to achieve potent combinatorial therapeutic effects and reduce their side effects have been pursued.

One of the most effective means for potent and low‐toxicity combine therapy is achieved via a nano‐scale drug delivery system by incorporating chemotherapeutic and targeted drugs through physical encapsulation or covalent bonding.^[^ ^15^ ^]^ The nano‐scale delivery system offers active or passive targeting of tumors and enables controlled release of drugs from the system within a tumor microenvironment (TME). Therefore, they can enhance drug distribution and reduce their toxic effects. Polymer‐based nano‐drug delivery systems have been showcased as the most promising one, and some of them have already progressed to clinical trials or clinical practice.^[^ ^16^ ^]^ Sugar‐derived polymers stand out from other polymers due to their hydrophilicity, cellular affinity, and degradability. In addition, multiple hydroxyl and aldehyde/ketone groups in sugar‐based polymers could form intermolecular hydrogen bonds, contributing to stable nano‐structures.^[^ ^17^ ^]^

The efficacy of drugs in the polymer‐based drug delivery system largely depends on the molecular structure, composition, and molecular weight of the polymer carrier.^[^ ^18^ ^]^ Advances in controlled polymerization and click chemistry have expanded the portfolio of polymer carriers, paving the way for novel sugar‐based carriers with specific chemical compositions and molecular structures.^[^ ^19^ ^]^ Our preliminary study suggests that polymers with branched structures offer better modifiability for both structure and function than linear polymers, thus they are promising templates for nano‐delivery systems.^[^ ^17^, ^20^ ^]^ Additionally, leveraging unique characteristic factors in the TME, including overexpressed enzymes, a low pH, and redox conditions, intelligent drug delivery systems, can achieve controlled drug release at tumor sites.^[^ ^21^ ^]^ Enzymes have advantages of their substrate specificity and mild reaction conditions. Response of nano‐delivery systems to overexpressed enzymes in the TME could minimize drug leakage and degradation during in vivo circulation, thus improving the drug delivery efficiency into tumor sites.

**Scheme 1:**
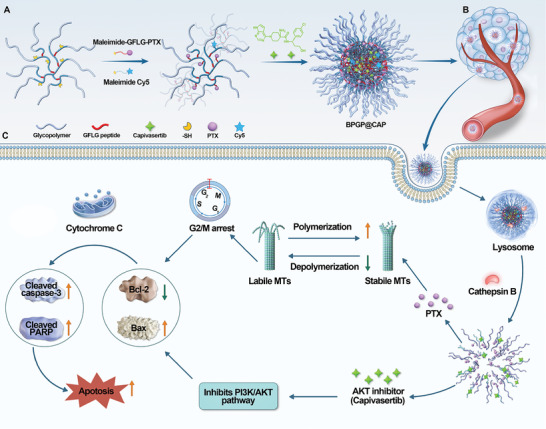
Schematic illustration of enzyme‐responsive branched glycopolymer‐based nanoassembly (BPGP@CAP) for synergistic antitumor therapy. A branched poly(LAEMA)‐GFLG‐PTX prodrug self‐assembles through hydrophobic‐hydrophilic interactions and this prodrug encapsulates CAP as an AKT inhibitor to form a stable nanoassembly, BPGP@CAP. Through the enhanced permeability and retention (EPR) effect, BPGP@CAP selectively accumulates at tumor tissues. Within tumor cells, overexpressed cathepsin B triggers specific cleavage of the GFLG peptide in the polymer structure, leading to carrier degradation and concomitant drug release. The released PTX and CAP synergistically exhibit antitumor effects by inducing apoptosis.

In this study, an innovative approach was proposed by utilizing 2‐lactobionamidoethylmethacrylamide (LAEMA) as a key building unit for branched glycopolymers to design and prepare an enzyme‐responsive nano‐drug delivery system for PTX and CAP. This system was engineered for precise targeting of tumor cells and controlled release of PTX and CAP. As illustrated in the **Scheme** **1**, LAEMA‐based branched polymer frameworks as a PTX prodrug were prepared through RAFT polymerization and thiol‐ene click reaction. These frameworks displayed self‐assembly characteristics, and they were used to encapsulate CAP during the self‐assembly process. Upon encountering tumor cells, the nano‐drug system underwent rapid disintegration due to the presence of overexpressed cathepsin B in the lysosomes to release PTX and CAP. This dual‐agent strategy exhibited notable synergy for GC: PTX hindered tumor cell proliferation by inhibiting mitosis and inducing apoptosis, and CAP restrained tumor cell growth and promoted apoptosis by suppressing the PI3K/AKT pathway. The encouraging therapeutic enhancement achieved through the nano‐delivery system underlines the potential of this dual‐drug co‐delivery approach as a promising therapeutic avenue for advancing GC treatment by combining chemotherapy and AKT inhibition.

## 2 Results and Discussion

### 2.1 Expression of AKT in GC Tissues and its Clinical Significance

**Figure 1:**
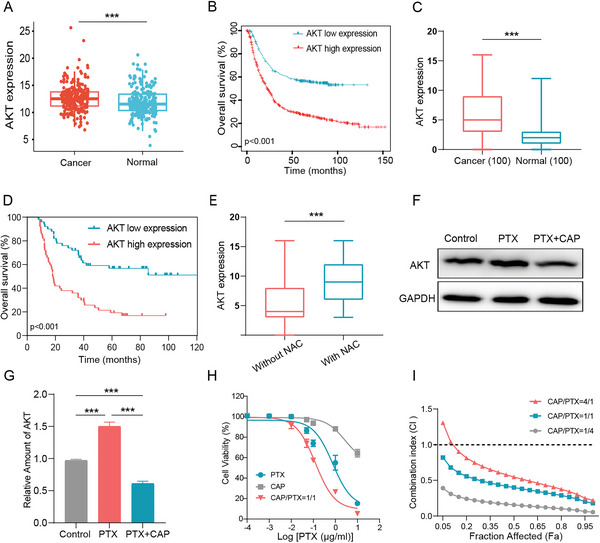
Clinical significance of AKT in gastric cancer (GC). A) The RNA expression of AKT in GC samples and normal gastric tissues was analyzed by utilizing the GEO cohort data. B) Overall survival rates of GC patients with a high or low level of AKT expression were analyzed through Kaplan‐Meier analysis. C) The AKT expression detected by IHC in GC samples and normal gastric tissues obtained from West China Hospital. D) Kaplan–Meier analysis of overall survival rates of GC patients based on the AKT expression level using the West China Hospital cohort data. E) IHC analysis of AKT expression in GC samples treated with and without neoadjuvant therapy (NAC) in West China Hospital. F) Western blot results of AKT expression in MFC cells treated with PTX (1 µg mL^‐1^) and PTX (1 µg mL^‐1^) + CAP (1 µg mL^‐1^). G) Semiquantitative analysis of the AKT protein level in different groups in Figure 1F. H) Cytotoxicity of PTX, CAP, and PTX + CAP toward MFC cells. I) Combination index (CI) of CAP and PTX for treating MFC cells at different CAP/PTX weight ratios (*n* = 5). Data is displayed as mean ± SD. A two‐sided unpaired Student's *t*‐test was employed for assessing statistical significance, ***p < 0.001.

Previous studies have validated abnormal activation of the PI3K/AKT signaling pathway in different types of tumors including GC could lead to unfavorable prognoses.^[^ ^22^ ^]^ The amplification of AKT mutations is believed to contribute to the activation of the PI3K/AKT signaling pathway, serving as one of the important factors responsible for this process.^[^ ^23^ ^]^ To reveal clinical significance of AKT expression in GC, analysis of AKT expression in GC tissues was performed and its relationship with prognosis using public databases assessed. The outcomes indicate a significantly elevated level of AKT expression in GC tissues in comparison to normal tissues (**Figure** **1**A). Furthermore, GC patients with a high level of AKT expression often have a significantly lower overall survival rate compared to those with a low level of AKT expression (Figure 1B). To validate this discovery, immunohistochemistry (IHC) analysis was conducted on clinical tissue samples obtained from the West China Hospital (Figure S1, Supporting Information). The analysis results (Figure 1C,D) are well aligned with the data in Figure 1A,B, corroborating the findings reported by Gu et al.^[^ ^24^ ^]^ Furthermore, previous research findings have demonstrated a strong correlation between the activation of the PI3K/AKT signaling pathway and drug resistance in various tumors.^[^ ^25^ ^]^

In this study, out of 100 GC patients, 25 received neoadjuvant chemotherapy, while 75 did not. IHC analysis supports a higher level of AKT expression in GC samples of patients who received neoadjuvant chemotherapy (*p* < 0.001) (Figure 1E). In addition, among 25 GC patients who underwent neoadjuvant chemotherapy, those with a lower level of AKT expression exhibit more pronounced tumor regression (Table S1, Supporting Information). Western blot results confirm increased expression of AKT in MFC cells after PTX treatment for 24 h (Figure 1F). These results suggest chemotherapy could boost the expression of AKT in MFC cells, and the expression of AKT may be related to the inefficacy of chemotherapy. It has been reported that an AKT inhibitor, CAP, can suppress the expression of AKT, leading to an enhanced sensitivity of tumor cells to chemotherapeutic drugs such as PTX.^[^ ^26^ ^]^ To demonstrate the impact of CAP on the cytotoxic effect of PTX, CAP combined with PTX was applied to treatmouse forestomach carcinoma (MFC) cells and western blot results confirm that AKT expression is inhibited (Figure 1F,G). The Cell Counting Kit‐8 (CCK‐8) assay was conducted to assess the cytotoxic effect of CAP combined with free PTX on MFC cells. The results demonstrate that the combination of CAP and PTX exhibits a significantly higher level of cytotoxicity compared to free PTX (Figure 1H), which indicates that the inhibition of AKT may be an effective approach to enhancing the efficacy of chemotherapy against GC.

The synergistic anti‐tumor effect of free CAP and PTX was then comprehensively evaluated. The cytotoxic effects of the combination of free CAP and PTX on MFC cells were assessed at various CAP/PTX weight ratios (CAP: PTX = 4:1, 1:1, and 1:4). The results reveal that almost all synergy indexes for the combination of CAP and PTX at different weight ratios are less than 1, suggesting a synergistic effect of CAP and PTX in killing MFC cells (Figure 1I). This demonstrates tremendous potential of the combine therapy of PTX and CAP in treating GC.

### 2.2 Fabrication and Characterizations of BPGP@CAP

In this study, we utilized controlled RAFT polymerization and efficient thiol‐ene reaction for designing and synthesizing the cathepsin B‐sensitive branched glycopolymer‐PTX prodrug. As shown in Scheme S1 (Supporting Information), a crosslinking agent, MA‐GFLG‐MA, and a small molecular prodrug, maleimide‐GFLG‐PTX, were first synthesized. Structural confirmation and purity assessment were performed using ^1^H NMR and liquid chromatography‐mass spectrometry (LC‐MS), and the experimental results are presented in Figures S2–S7 (Supporting Information). After successful preparation of polymerizable monomers and functionalized small molecules, RAFT polymerization was conducted to produce a high‐molecular‐weight chain transfer agent, poly(LAEMA)‐CTA, illustrated in Scheme S2A (Supporting Information). An approximate value of 28 kDa is obtained from molecular weight calculation via ^1^H NMR and the repeating LAEMA units are ≈61 (Figure S8, Supporting Information). Gel permeation chromatography (GPC) analysis reveals a PDI of ≈1.06 for the chain transfer agent (Figure S13, Supporting Information), indicative of a narrow molecular weight distribution.

**Figure 2:**
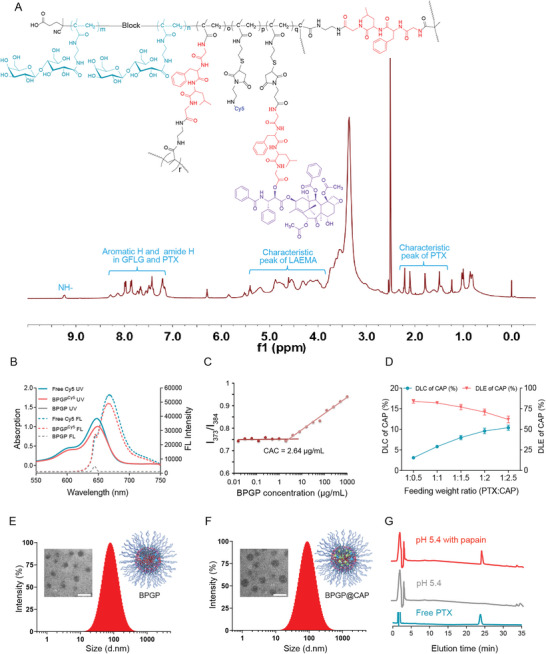
Characterizations of physicochemical properties of BPGP and BPGP@CAP. A) ^1^H NMR of the branched poly(LAEMA)‐GFLG‐PTX prodrug in DMSO‐*d6*. B) UV–*vis* and fluorescence spectra of the branched poly(LAEMA)‐GFLG‐PTX prodrug (dissolved in DMSO) with or without Cy5 labeling. The characteristic UV–vis absorption peak of Cy5 appears at 648 nm, and the fluorescence characteristic peak at 667 nm (Ex = 647 nm). C) CAC of the branched poly(LAEMA)‐GFLG‐PTX prodrug. D) The drug loading content (DLC) and the drug loading efficiency (DLE) of CAP in BPGP at various weight ratios of PTX to CAP. E,F) Hydrodynamic diameters and representative TEM images of BPGP and BPGP@CAP, respectively. Scale bars = 100 nm. G) HPLC chromatograms of poly (LAEMA)‐GFLG‐PTX with or without papain, and free PTX as a control.

Subsequently, co‐polymerization of poly(LAEMA)‐CTA, MA‐GFLG‐MA, MA‐PySS, and LAEMA yields an intermediate polymer with a branched architecture (branched poly(LAEMA)‐GFLG‐PySS). The ^1^H NMR spectrum of the intermediate polymer displays significant characteristic peaks of pyridine at 7.22 ppm, 7.74 ppm, and 8.31 ppm, indicating successful introduction of MA‐PySS into the polymer structure (Figure S9, Supporting Information). After deprotection treatment, the characteristic peak of pyridine in the polymer disappears from the ^1^H NMR spectrum, while a characteristic peak of the benzene ring in GFLG is observed at 7.29 ppm (Figure S10, Supporting Information). The as‐prepared intermediate polymer was sequentially conjugated with maleimide‐Cy5 and maleimide GFLG PTX, resulting in a product of a branched polymer prodrug, poly(LAEMA^Cy5^)‐GFLG‐PTX (termed as BPGP). Structural confirmation of the product is attained through its ^1^H NMR (**Figure** **2**A). Notably, distinct peaks corresponding to PTX and GFLG are not observed in the ^1^H NMR spectrum of BPGP in D_2_O, which is distinctly different from the spectrum of BPGP in DMSO‐*d6*. This disparity suggests BPGP may experience self‐assembly in an aqueous solution (Figure S11 and S12, Supporting Information). As shown in Figure 2B, compared with unlabeled branched polymer prodrugs, the characteristic peak of Cy5 can be observed in both ultraviolet visible (UV‐*vis*) and fluorescence spectra, and there is no significant change in the wavelength of the characteristic peak compared to free Cy5, indicating that the optical properties of Cy5 remain after it was covalently coupled to the polymer.

Due to the presence of hydrophilic segments and hydrophobic drugs in the branched polymer prodrug, the prodrug could self‐assemble into nanoparticles through hydrophilic hydrophobic interactions. We used pyrene as a fluorescence probe to detect its critical micelle concentration (CAC), and its CAC value is found to be ≈2.64 µg mL^−1^, which indicates that the polymer prodrug possesses a remarkable self‐assembly ability (Figure 2C). Subsequently, we assessed the potential of the PTX prodrug as a polymeric nanocarrier system for drug delivery. To realize the synergy of chemotherapy and molecular targeted therapy, we utilized a solvent evaporation technique to encapsulate CAP, an AKT inhibitor, into the glycopolymer‐PTX prodrug, resulting in a nanoassembly, BPGP@CAP. Initially, the nanoassembly was fabricated at different PTX‐to‐CAP feed ratios to assess the encapsulation capability of the polymer‐PTX prodrug for CAP. As shown in Figure 2D, with an increase in the CAP feed weight, the drug loading of CAP within the nanoassembly progressively augments, signifying a great encapsulation capacity of the nanoassembly. However, it's worth noting that as the drug loading increases, the encapsulation efficiency of the nanoassembly exhibits a gradual decline. Notably, when the weight ratio of PTX to CAP drops below 1:1.5, the encapsulation efficiency of the nanoassembly experiences a substantial reduction. Consequently, the nanoassembly synthesized at a PTX‐to‐CAP weight ratio of 1:1.5 is chosen for subsequent experimental uses. The results of HPLC analysis reveal that 7.5 wt.% of PTX and 8.0 wt.% of CAP are in the nanoassembly of BPGP@CAP. As shown in Figure 2E,F, DLS measurements confirm that the hydrodynamic diameter of BPGP is ≈78.83 ± 0.35 nm, and upon encapsulation of CAP, a slight increase in the hydrodynamic size is observed (90.12 ± 0.36 nm). TEM images exhibit a relatively uniform size distribution for both BPGP and BPGP@CAP. Although encapsulation of CAP leads to a marginal size augmentation, the morphology of the nanoassembly remains unchanged. Leveraging the presence of GFLG in the polymer structure, the drug release behavior from the polymer prodrug was probed in a simulated tumor intracellular microenvironment. Since papain and cathepsin B share the same catalytic site, papain was selected to mimic cathepsin B in the tumor microenvironment. As depicted in the Figure 2G, under conditions without papain catalysis, the polymer prodrug retains stability with negligible drug release. Conversely, under enzymatic catalysis, the polymer prodrug effectively releases the loaded drug. These findings suggest the polymer prodrug could be used for stable in vivo drug delivery and controlled drug release at tumor sites.

### 2.3 Cellular Uptake of BPGP@CAP by MFC Cells

**Figure 3:**
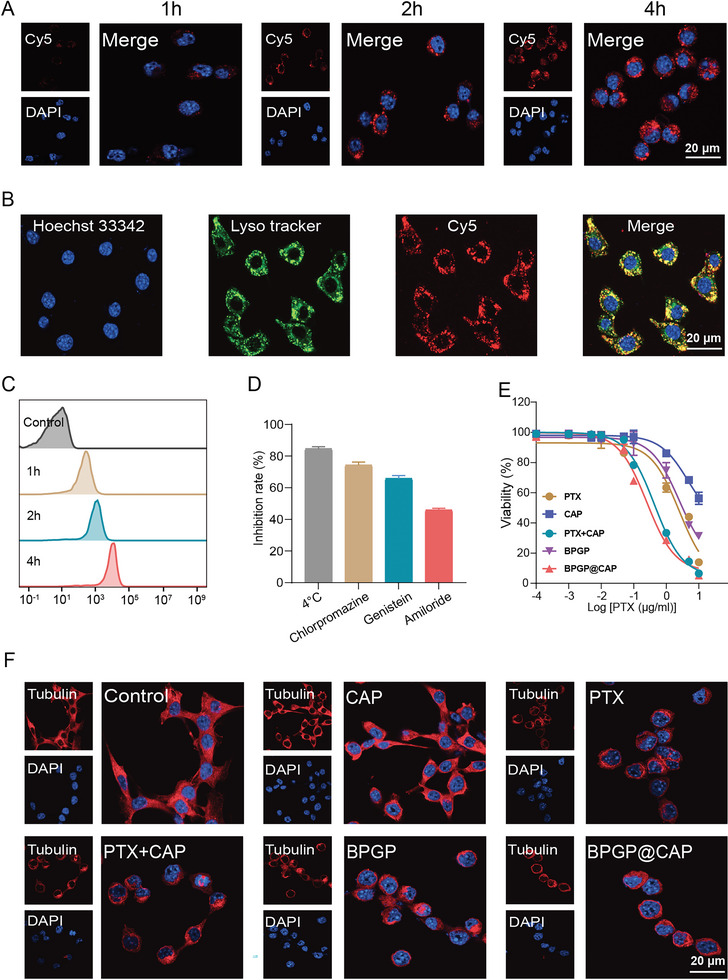
Cellular uptake and cytotoxicity of BPGP@CAP in vitro. A) CLSM images of MFC cells exposed to Cy5‐labelled BPGP@CAP (red: Cy5; blue: nuclei). Scale bar = 20 µm. B) Colocalization of lysosomes and BPGP@CAP in MFC cells. Lyso‐Tracker was used to label and visualize the lysosomes (green), while Hoechst 33342 was employed to stain the nuclei (blue). Scale bar = 20 µm. C) Mean fluorescence intensity (MFI) of MFC cells after exposure to Cy5‐labelled BPGP@CAP for 1 h, 2 h, and 4 h from flow cytometry analysis. D) The inhibitory rate of cellular uptake of BPGP@CAP by MFC cells treated with different inhibitors or at a low temperature. E) Viabilities of MFC cells after treatment with PTX, CAP, PTX + CAP, BPGP and BPGP@CAP. F) Microtubule aggregation in MFC cells induced by various treatments for 24 h. Tubulin Tracker was utilized to stain and detect tubulin (red), while DAPI was employed for staining the nuclei (blue). Scale bar = 20 µm.

Cellular uptake of polymeric drug delivery systems by tumor cells is a crucial step to exert a drug antitumor effect. The uptake of Cy5‐labeled BPGP@CAP into MFC cells was evaluated using CLSM and flow cytometry techniques. By observing the fluorescence signal of Cy5 in MFC cells after incubation with Cy5‐labeled BPGP@CAP at different durations through CLSM, it is evident that with an increase in the incubation time, the red fluorescence signal from Cy5 gradually accumulates and becomes intensified in the cytoplasm of MFC cells (**Figure** **3**A; Figure S14, Supporting Information). Flow cytometry data for uptake of Cy5‐labeled BPGP@CAP by MFC cells is in agreement with the CLSM images (Figure 3C; Figure S15, Supporting Information). These results indicate that BPGP@CAP could be efficiently ingested by MFC cells, and this uptake process is time‐dependent.

To investigate the endocytic pathways of BPGP@CAP into MFC cells, flow cytometry was employed to detect the uptake of BPGP@CAP by MFC cells at a low temperature. The results show that the uptake of BPGP@CAP by MFC cells is significantly inhibited at an inhibition rate of 80.5%, indicating that the internalization of BPGP@CAP is an energy‐dependent process. Furthermore, upon incubating inhibitors with MFC cells, flow cytometry data demonstrate that chlorpromazine, genistein, and amiloride inhibit cellular uptake of BPGP@CAP by 70.2%, 65.4%, and 44.5% respectively. This suggests that clathrin‐mediated, caveolin‐mediated, and icropinocytosis‐mediated endocytic pathways may be involved in the uptake of BPGP@CAP (Figure 3D; Figure S16, Supporting Information). Moreover, since the lysosomal cathepsin B can cleave the GFLG linker to release PTX,^[^ ^27^ ^]^ we investigated whether BPGP@CAP would be transported to the lysosomes for degradation after cellular uptake. Through lysosomal colocalization experiments (Figure 3B), we observe a high overlapping level of the fluorescence signal for BPGP@CAP and lysosomes, suggesting BPGP@CAP may land at lysosomes before it is disintegrated by lysosomal cathepsin B. Combined with the findings from in vitro drug release experiments, it is confirmed that BPGP@CAP can be internalized by MFC cells and release drugs stimulated by lysosomal cathepsin B.

### 2.4 The Toxicity of BPGP@CAP on MFC Cells

To assess the toxicity of BPGP@CAP, we conducted CCK‐8 assays to measure the viability of MFC cells subjected to various treatment groups. The results indicate that after the treatment of BPGP@CAP, MFC cells viability significantly decreases. BPGP@CAP has an IC_50_ of 0.253 µg mL^−1^ in comparison to free PTX with an IC_50_ of 2.51 µg mL^−1^ and CAP with an IC_50_ of 4.74 µg mL^−1^. Furthermore, the cell viability in the group with the treatment of BPGP@CAP is similar to that in the group treated with free PTX+CAP with an IC_50_ of 0.43 µg mL^−1^, whereas the cell viability in the group treated with BPGP is comparable to that in the group exposed to free PTX, and the IC_50_ value for BPGP and free PTX is 2.52 µg mL^−1^ and 2.51 µg mL^−1^, respectively (Figure 3E). These findings suggest that BPGP@CAP could effectively release PTX and CAP after disintegration of the nanoassembly structure in the lysosomes, thus exerting an equivalent antitumor effect to a mixture of free PTX and CAP. Additionally, both the groups treated with BPGP@CAP and PTX + CAP exhibit a significantly higher cytotoxic effect on MFC cells than the groups exposed to BPGP and free PTX, indicating that the combination of CAP and PTX displays a synergistic action on MFC cells.

Microtubules, which are essential cytoskeletal filaments within cells, have a critical function in mitosis and intracellular vesicle transport.^[^ ^28^ ^]^ It is well‐established that PTX can enhance the polymerization of microtubules, hinder depolymerization, and suppress mitosis of tumor cells.^[^ ^29^ ^]^ In order to assess the impact of PTX derived from BPGP@CAP on microtubules, we used CLSM to observe morphological changes of microtubules in the MFC cells after various treatments. Microtubules are evenly distributed in the cytoplasm of MFC cells in the control and CAP‐treated groups. However, pronounced aggregation of microtubules is found around the cell nucleus in the groups exposed to PTX and PTX+CAP. Similar morphological changes of microtubules are observed in the groups treated with BPGP and BPGP@CAP (Figure 3F). Furthermore, in the control and CAP‐treated groups, the nuclei of MFC cells appear intact, whereas an abnormal nuclear morphology and a multinucleated structure are found in the groups treated with PTX, PTX + CAP, BPGP and BPGP@CAP, indicating that PTX released from BPGP@CAP exerts a similar effect on microtubules as free PTX.

Furthermore, microfilaments, which are composed of actin proteins, are integral constituents of the cellular cytoskeleton. Through CLSM, the influence of BPGP@CAP on microfilaments of MFC cells was examined. Our results indicate that actin aggregates into clusters or dots near the cell membrane in the BPGP@CAP‐treated group (Figure S17, Supporting Information), leading to impaired microfilaments and reduced cell viability.

**Figure 4:**
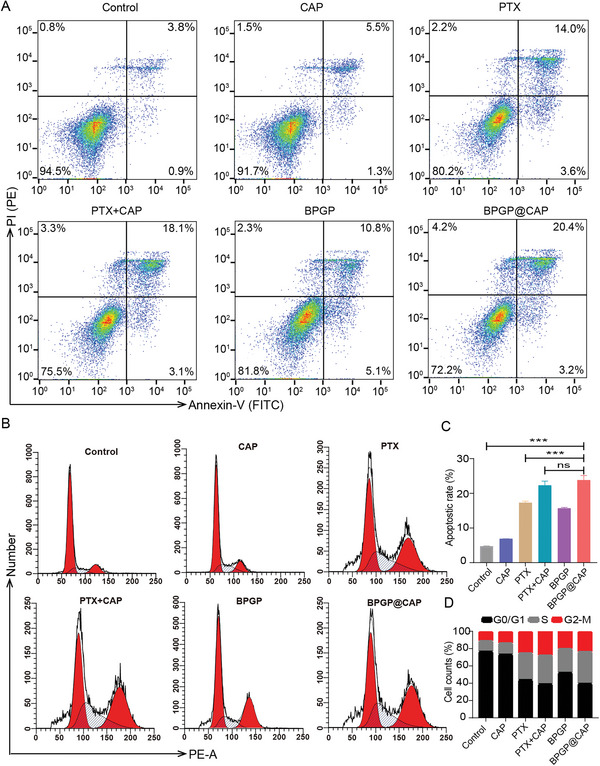
Analysis of apoptosis and cell cycle arrest in MFC cells after exposure to various treatments through flow cytometry. A,C) Percentages of apoptotic MFC cells after exposure to PTX, CAP, PTX + CAP, BPGP, and BPGP@CAP for 48 h (*n* = 3). B,D) Cell cycle distribution of MFC cells after exposure to PTX, CAP, PTX + CAP, BPGP and BPGP@CAP for 48 h (*n* = 3). Data is displayed as mean ± SD. A two‐sided unpaired Student's *t*‐test was employed for assessing statistical significance, **p < 0.01, ***p < 0.001 and ns for *p* > 0.05.

Moreover, we utilized flow cytometry to evaluate the impact of BPGP@CAP on the apoptosis of MFC cells. The findings indicate that the percentage of apoptotic cells in the groups exposed to BPGP@CAP (23.8%), BPGP (15.7%), PTX (17.3%), and PTX+CAP (22.3%) is significantly higher than that in the control group (4.7%) (**Figure** **4**A,C). Additionally, no significant difference is found in the percentage of apoptotic cells between the groups exposed to BPGP@CAP and PTX + CAP. Furthermore, MFC cells have a significantly higher apoptotic percentage after treatment with BPGP@CAP and PTX + CAP than those treated with PTX and CAP, further supporting their synergistic action of inducting apoptosis of MFC cells.

It is widely recognized that PTX exerts its effects on microtubules, causing inhibition of cell mitosis and resulting in G2/M cell cycle arrest.^[^ ^30^ ^]^ We assessed the influence of PTX released from BPGP@CAP on the cell cycle distribution of MFC cells. As presented in Figure 4B,D, the percentages of cells in the G2/M phase in the groups treated with PTX (25.1%), BPGP (20.2%), PTX + CAP (27.8%), and BPGP@CAP (23.6%) are significantly higher than that in the control group (11.0%). Conversely, the proportions cells in the G0/G1 phase in the groups treated with PTX (43.9%), BPGP (52.2%), PTX + CAP (39.0%), and BPGP@CAP (39.6%) are significantly lower than that in the control group (76.6%). These findings support that PTX released from BPGP and BPGP@CAP can lead to cell cycle arrest in the G2/M phase. These results further confirm that BPGP@CAP can effectively releases PTX in the tumor cells, thereby displaying a similar antitumor mechanism of action as free PTX.

### 2.5 Mechanism Study on the Synergistic Effect of PTX and CAP

**Figure 5:**
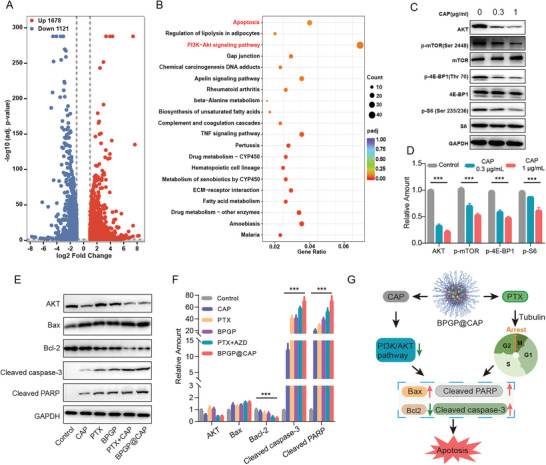
Mechanisms underlying synergistic effect of PTX and CAP for anticancer treatment. A) Volcano plots of DEGs in BPGP@CAP‐treated MFC cells compared with the control group. B) Top 20 enriched KEGG pathways from DEGs in MFC cells exposed to BPGP@CAP compared to the control cells. C, D) Western blotting analysis of proteins associated with the PI3K/AKT signal pathway in MFC cells treated with CAP, including AKT, p‐mTOR, p‐4E‐BP1, and p‐S6. The proteins in the control group are significantly lower than those in the groups treated with CAP at 0.3 µg mL^‐1^ and 1.0 µg mL^‐1^, and the P‐values are less than 0.001. E,F) Western blot results of proteins associated with apoptosis in MFC cells after exposure to PTX, CAP, PTX + CAP, BPGP, and BPGP@CAP, including Bax, Bcl‐2, cleaved‐caspase‐3 and cleavedPARP. The proteins in the control group are significantly lower than those in the groups treated with PTX, CAP, PTX + CAP, BPGP, and BPGP@CAP, and the P‐values are less than 0.001. G) Schematic diagram for the mechanisms for the anticancer effect by the combine therapy with PTX and CAP against GC. Data were displayed as mean ± SD. A two‐sided unpaired Student's *t*‐test was employed for assessing statistical significance, ***p < 0.001.

Our in vitro experimental results support the existence of a synergistic effect between PTX and CAP to eliminate MFC cells. To explore the underlying mechanisms behind this synergy, we conducted transcriptome sequencing on MFC cells exposed to BPGP@CAP and compared them with the control group. We detected 2799 differentially expressed genes (DEGs) between BPGP@CAP and control groups. Out of these DEGs, 1678 are upregulated and 1121 downregulated in the BPGP@CAP‐treated group (**Figure** **5**A; Figure S18, Supporting Information). Subsequently, Kyoto Encyclopedia of Genes and Genomes (KEGG) pathway enrichment analysis was employed to uncover the biological pathways impacted by these DEGs. The results suggest that the pathways for apoptosis and PI3K/AKT signaling are significantly upregulated, which indicates that the mechanism underlying the synergistic antitumor effects of PTX and CAP in BPGP@CAP may be related to the PI3K/AKT signaling pathway and apoptosis (Figure 5B). It has been evidenced that the PI3K/AKT signaling pathway can regulate apoptosis by modulating the phosphorylation of Bax and Bad and the regulation of their expression levels, the release of cytochrome C, and the activation of caspase ‐ 3 and 9, thus affecting cell survival.^[^ ^31^ ^]^ Western blot results confirm that CAP inhibits the expression of the PI3K/AKT pathway‐related proteins such as AKT, p‐mTOR, p‐S6, and p‐4EBP1 (Figure 5C), indicating that CAP can suppress the PI3K/AKT signaling pathway.

Furthermore, western blotting was employed to assess the expression of apoptosis‐related proteins, such as cleaved PARP, cleaved caspase‐3, Bax, and Bcl‐2, in various treatment groups. As showed in Figure 5E,F, the expression of Bax, cleaved caspase‐3, and cleaved PARP is upregulated in MFC cells after treatment with CAP, which might be credited to apoptosis after the inhibition of AKT. Furthermore, it is known that PTX can induce tumor cell apoptosis by downregulation of Bcl‐2 and upregulation of Bax and cleaved caspase‐3.^[^ ^32^ ^]^ Our results confirm that the expression levels of Bax, cleaved caspase‐3, and cleaved PARP were elevate, while Bcl‐2 is downregulated in the MFC cells with treatment of PTX and BPGP. In addition, when MFC was treated with the combination of free PTX and CAP or BPGP@CAP, more pronounced changes are observed in the expression of proteins including Bax, cleaved caspase −3, and cleaved PARP. Interestingly, western blot results reveal that the effect of CAP on downregulating the BCL‐2 protein expression is not pronounced as PTX, suggesting that the downregulation of Bcl‐2 may be predominantly induced by PTX, while no apparent correlation is seen between the downregulation of Bcl‐2 and the inhibition of AKT expression. However, the combination of PTX and CAP can induce more significant downregulation of Bcl‐2 compared to their individual effects, which is in agreement with a previous study.^[^ ^26^ ^]^ It may be explained by that CAP could significantly sensitize MFC cells to PTX‐induced apoptosis, resulting in more prominent downregulation of Bcl‐2.

The combination of PTX with CAP exerts synergistic effects on MFC cells via the modulation of pro and anti‐apoptotic genes, such as downregulation of Bcl‐2 and upregulation of Bax, cleaved caspase‐3, and cleaved PARP. This could be the underlying mechanism for synergistic promotion of apoptosis by PTX and CAP (Figure 5G).

### 2.6 Antitumor Effect of BPGP@CAP In Vivo

#### 2.6.1 *Distribution of BPGP@CAP* In Vivo

**Figure 6:**
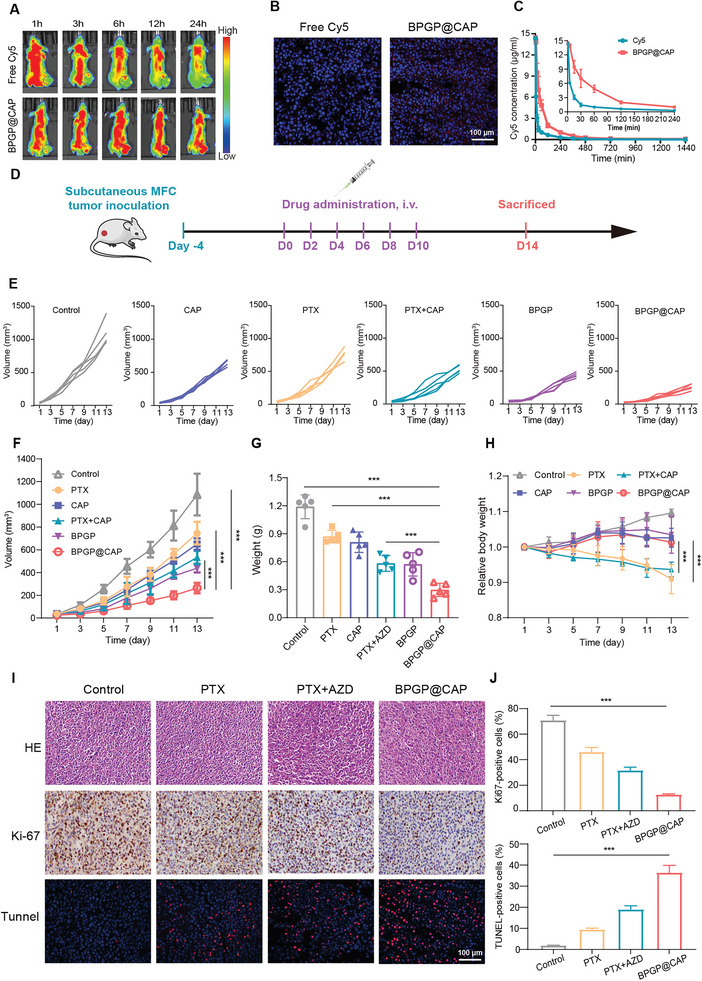
Biodistribution and tumor growth inhibition of BPGP@CAP in vivo. A) Fluorescent images of the mice with MFC tumors exposed to Cy5‐labelled BPGP@CAP and free Cy5 at different time‐points. B) Immunofluorescence staining images of tumor tissues obtained from the mice treated with free Cy5 and Cy5‐labelled BPGP@CAP for 24 h. Red for Cy5‐labeled BPGP@CAP or free Cy5, and blue for DAPI‐stained cell nuclei. Scale bar = 100 µm. C) Pharmacokinetic curves of the Cy5 concentration after intravenous injection of Cy5‐labelled BPGP@CAP and free Cy5, respectively (*n* = 5). D) Establishment of subcutaneous GC MFC tumor models and therapeutic treatment schedule. E) Growth curves of the tumors in individual mice across different treatment groups (*n* = 5). F) Average tumor growth curves in different treatments groups (*n* = 5). G) Tumor weights of the mice with tumors in different treatment groups (*n* = 5). H) Body weights of the mice with tumors after different treatments (*n* = 5). The statistical difference is obtained from the body weights of the BPGP@CAP‐treated group in comparison with those of the PTX‐treated group and the PTX+CAP‐treated group. I) H&E, TUNEL and Ki67 staining images for tumor tissues from the mice after different treatments (scale bar = 100 µm). J) The proportion of TUNEL and Ki67‐positive cells in the tumors after different treatments. The proportion of TUNEL and Ki67‐positive cells of the BPGP@CAP‐treated group is compared with that in the control group, the PTX‐treated group and the PTX+CAP‐treated group. Data are displayed as mean ± SD. A two‐sided unpaired Student's t‐test was employed for assessing statistical significance, ***p < 0.001.

The accumulation of BPGP@CAP at the site of transplanted tumor plays a critical role in its antitumor efficacy in vivo. Mounting evidence has suggested that nanoparticles exhibit the enhanced permeability and retention (EPR) effect, which can extend the residence time and increase the concentration of drugs in tumors, thus achieving passive drug targeting of tumor cells.^[^ ^33^ ^]^ The characterization results of BPGP@CAP indicate that it possesses a nanoscale size for the EPR effect. To confirm the accumulation of BPGP@CAP in tumor tissues via the EPR effect, we utilized a fluorescence imaging technique to evaluate the distribution of BPGP@CAP in the mice with MFC tumors. By administering Cy5‐labeled BPGP@CAP and free Cy5 intravenously to the tumor‐bearing nude mice, we assessed the distribution of BPGP@CAP at various time intervals using IVIS spectroscopy. The findings reveal that the fluorescence intensity of the tumor in the BPGP@CAP‐treated group is stronger than that in the free Cy5‐treated group as time elapses (**Figure** **6**A; Figure S19, Supporting Information). Specifically, after 24 h, the fluorescence intensity of the tumor in the BPGP@CAP‐treated group still remains strong, while faint fluorescence signal is detected in the tumor of the free Cy5‐treated group. This indicates that BPGP@CAP can accumulate and retain in the tumor site for an extended period. Furthermore, tumor tissues were collected at 24 h post‐injection and visualized using CLSM. The images demonstrate that tumor tissues of the mice after the treatment of free Cy5 show undetectable fluorescence signal, whereas tumor tissues of the mice after the treatment of BPGP@CAP exhibit significantly strong fluorescence intensity (Figure 6B). The in vivo imaging results confirm the enrichment of BPGP@CAP in tumor tissues.

Furthermore, pharmacokinetic analysis in vivo reveals a slower decrease in the Cy5 concentration in the blood of the mice exposed to BPGP@CAP compared to that in the group exposed to free Cy5, and the half‐life of BPGP@CAP and free Cy5 was 488.49 and 160.85 min, respectively (Figure 6C; Table S2, Supporting Information). The results might be ascribed to a high molecular weight of BPGP@CAP and a prolonged circulation time which may help enhance passive accumulation of BPGP@CAP at the tumor site, thereby improving its therapeutic efficacy against tumors.

#### 2.6.2 Antitumor Effect of BPGP@CAP

The mice, which were subcutaneously injected with MFC cells, were divided randomly into six groups, each consisting of 5 mice. The control group were given intravenous injections of saline, while the other groups were intravenously injected with PTX, CAP, PTX + CAP, BPGP, and BPGP@CAP, respectively. The concentrations of PTX remain consistent at 8 mg kg^−1^ for each mouse. The treatment schedule is illustrated in Figure 6D. The volume of the transplanted tumor and the body weight of each treated mouse were regularly monitored and recorded. The results reveal that the control group has the fastest tumor growth rate, while the BPGP@CAP‐treated group displays the slowest tumor growth (Figure 6E). After the treatment, the tumor volume of the control group is 1.45, 1.67, 2.04, 2.48, and 4.13 times that of the group treated with PTX, CAP, PTX+CAP, BPGP, and BPGP@CAP, respectively. The tumor inhibition rates, based on tumor volume measurements, are found to be 31.0%, 40.1%, 51.0%, 59.7%, and 75.8% after treatment with PTX, CAP, PTX + CAP, BPGP, and BPGP@CAP, respectively. In addition, the tumor volumes in the groups with the treatment of free PTX and PTX + CAP are 2.85 times and 2.02 times that of the BPGP@CAP‐treated group, respectively (Figure 6F). The tumor volumes of the transplanted tumors harvested from each group are well aligned with those observed in vivo (Figure S20A, Supporting Information). Comparison of the transplant tumor masses in the treatment groups reveals that the tumor mass harvested from the BPGP@CAP‐treated mice (0.28 g) is significantly lighter than that of the free control group (1.20 g), the PTX‐treated group (0.84 g), and the PTX + ACP‐treated group (0.57 g) (Figure 6G). The tumor inhibition rates, based on tumor mass measurements, are estimated to be 30.2%, 39.1%, 49.6%, 58.6%, and 75.7% for PTX, CAP, PTX + CAP, BPGP, and BPGP@CAP, respectively (Figure S20B, Supporting Information). The tumor inhibition rates based on tumor mass measurements are similar to those based on tumor volume measurements in vivo for CAP and/or PTX‐derived formulations. The difference in the tumor inhibition rates among BPGP@CAP and other groups may result from a synergistic effect of PTX and CAP, enrichment of BPGP@CAP in tumor tissues and prolonged circulation in the blood through a nanoscale drug delivery system.

The antitumor effect of BPGP@CAP was further verified by IHC analysis of tumor tissues. TUNEL assays of the harvested tumors indicate TUNEL‐positive tumor cells in the BPGP@CAP‐treated group are the most populous, confirming that the treatment of BPGP@CAP results in more apoptotic cells compared with other groups (Figure 6I,J). Furthermore, the mice treated with BPGP@CAP show a significant reduction in the population of Ki67‐positive tumor cells with comparison to the other groups. This observation suggests that the administration of BPGP@CAP leads to a remarkable suppression of tumor cell proliferation (Figure 6I,J), which confirms synergistic inhibition of tumor growth by both agents in vivo. These findings support the excellent anti‐tumor effect of BPGP@CAP.

Additionally, while the administration of free PTX and PTX + CAP effectively suppresses the growth of transplanted tumors in the mice, it is accompanied by a decrease in the body weight ranging from 9.1% to 6.3% after each administration (Figure 6H). This indicates the presence of systemic toxicity associated with free PTX. In contrast, no significant changes in the body weight are found in the BPGP and BPGP@CAP‐treated groups, suggesting that the toxicity of PTX could be remarkably reduced after PTX is delivered via a polymer prodrug. In addition, we conducted H&E staining on the major organs of the mice and analyzed the change of hematological parameters including white blood cells (WBC), red blood cells (RBC), platelets (PLT), alanine aminotransferase (ALT), aspartate aminotransferase (AST), blood urea nitrogen (BUN), and creatinine (CREA) to evaluate the toxicity of BPGP@CAP. The H&E staining images indicate no significant alterations in the major organs, including the heart, liver, spleen, lung, and kidney, across all treatment groups (Figure S21, Supporting Information). However, compared to the control group with a WBC count of 8.20×10^9^/L, the WBC count of the mice is found to be 3.19 × 10^9^ per L, 3.28 × 10^9^ per L, 5.76 × 10^9^ per L and 5.69 × 10^9^ per L in the group treated free PTX, PTX + CAP, BPGP, and BPGP@CAP, respectively. The most pronounced decreases in the WBC number are seen in the groups treated with PTX and PTX + CAP. Furthermore, when compared to the control group where the ALT level is 21.0 U L^−1^, there is no significant change in the ALT level in the BPGP+CAP‐treated group (22.0 U L^−1^), while a distinctive increase is seen in the PTX group (71.7 U L^−1^) and the PTX+CAP group (63.3 U L^−1^) (Figure S22, Supporting Information). These results indicate that systemic administration of free PTX or PTX + CAP can cause hematopoietic system toxicity and liver toxicity, while the use of BPGP@CAP to deliver PTX and CAP can significantly reduce their toxicity.

## 3 Conclusions

In this study, a cathepsin B‐responsive drug delivery system has been established for co‐delivery of PTX and CAP using controlled RAFT polymerization and efficient click chemistry, aiming to achieve a synergistic therapeutic outcome from the combine therapy of chemotherapy and targeted therapy for GC. The BPGP@CAP nanoassembly enables targeted delivery of both PTX and CAP into the tumor site through the EPR effect, and precise release of PTX and CAP from BPGP@CAP after intelligently responding to overexpressed cathepsin B in the lysosomes of tumor cells. The released CAP suppresses the expression of AKT and its downstream PI3K/AKT pathway, and it synergizes with the released PTX to exert an enhanced anti‐tumor effect. Meanwhile, the toxicity of PTX and CAP is significantly reduced after their delivery in this glycopolymer‐based drug delivery system. Therefore, this enzyme‐responsive nanomedicine based on glycopolymer prodrugs could be utilized for co‐delivery of multiple therapeutic agents, providing a novel approach for GC treatment.

## 4 Experimental Section

### Materials

CAP and PTX were obtained from Selleck Chemicals (Shanghai, China). Maleimide cyanine5 was acquired from Confluore Biotech CO. Ltd. (Xian, China). Chlorpromazine hydrochloride, amiloride hydrochloride, and dynasore were bought from APExBIO (Houston, USA). A terminal deoxynucleotidyl transferase‐mediated deoxyuridine triphosphate nick end labeling (TUNEL) Apoptosis Assay Kit was bought from Promega (Beijing, China). Hoechst 33342 and Cell Apoptosis Kit were purchased from Yeasen (Shanghai, China). Actin‐Tracker Green, Tubulin‐Tracker Red and Lyso‐Tracter Green were purchased from Beyotime (Shanghai, China). CCK‐8 was bought from MCE (Shanghai, China).

### Synthesis of LAEMA‐Based Branched Polymeric Prodrug

*Synthesis of Poly (LAEMA)‐CTA*: 4‐cyano‐4‐((phenylcarbonothioyl)thio)pentanoic acid (chain transfer agent, CTA) (18.6 mg, 66.7 µmol) and LAEMA (2001.6 mg, 4.275 mmol) were mixed within a small‐necked 25 mL flask. The flask was subjected to argon purging, and the process was repeated thrice. Under an ice bath condition, an initiator solution containing VA044 (7.2 mg, 22.2 µmol) in a solvent mixture of water and methanol (H_2_O/CH_3_OH = 3:2, 9.1 mL) was introduced into the flask via syringe injection. The reaction mixture was thereafter subjected to bubbling with a continuous stream of argon for a duration of 30 min. The flask was then placed in a light‐protected environment at 45 °C and the reaction continued for 20 h. After the polymerization was completed, the reaction was quenched using liquid nitrogen. The reaction mixture underwent dialysis against deionized water (MWCO 3500 Da) for 1.5 days and was subsequently freeze‐dried to yield the product poly (LAEMA)‐CTA with a 78% yield.

*Synthesis of Branched Poly (LAEMA)‐GFLG‐PySS*: To synthesize the branched polymer poly(LAEMA)‐GFLG‐PySS, poly(LAEMA)‐CTA (270 mg), a monomer MA‐PySS (29 mg, 113 µmol), LAEMA (107 mg, 229 µmol), and a crosslinker MA‐GFLG‐MA (15 mg, 26 µmol) were mixed in a small‐necked 5 mL round‐bottom flask. The flask was purged with argon gas three times. Under an ice bath condition, a solution containing the initiator, VA044 (1.62 mg), in a solvent mixture of water and dimethylformamide (H_2_O/DMF = 2:7, 1.9 mL) was introduced into the flask via syringe injection. The reaction mixture was continuously bubbled with argon for 30 min. The flask was then placed in a light‐protected environment at 45 °C and the reaction continued for 24 h. Upon completion of the reaction, the reaction was quenched using liquid nitrogen. The reaction mixture underwent dialysis against deionized water (MWCO 3500 Da) for a duration of 1.5 days, followed by freeze‐drying to obtain the final product, branched poly(LAEMA)‐GFLG‐PySS, with a yield of 67%.

*Synthesis of Branched Poly(LAEMA)‐GFLG‐PTX*: In a round‐bottom flask, 200 mg of branched poly(LAEMA)‐GFLG‐PySS was accurately weighed and dissolved in 8 mL of a mixture of DMSO:H_2_O (4:1, v/v). After complete dissolution, 30 mg of dithiothreitol (DTT) was introduced, and the reaction mixture underwent 12 h reaction at room temperature. Subsequently, the reaction mixture was subjected to dialysis to remove organic solvents, followed by freeze‐drying to yield the product, branched poly(LAEMA)‐GFLG‐SH.

Branched poly (LAEMA)‐GFLG‐SH(146 mg) was carefully weighed and dissolved in 8 mL of a mixture of DMSO:H_2_O (4:1, v/v) in a round‐bottom flask. After complete dissolution, 1.4 mg of maleimide‐Cy5 dissolved in DMSO was added to the reaction mixture. The reaction proceeded overnight at room temperature. 48.7 mg of maleimide‐GFLG‐PTX was then introduced into the reaction mixture, and the reaction continued at room temperature for an additional 12 h. After the completion of the reaction, the mixture was sequentially dialyzed with DMF and deionized water (MWCO = 3500 Da). The resulting product was collected, filtered through a membrane, and subjected to freeze‐drying, resulting in the formation of branched poly(LAEMA^Cy5^)‐GFLG‐PTX. The drug loading of PTX in the polymer was determined through HPLC analysis.

### Physicochemical Characterizations of BPGP and BPGP@CAP

The assessment of the CAC of BPGP was accomplished with the aid of pyrene as a fluorescence agent. Herein, 200 µL of a pyrene‐acetone solution (0.67 × 10^−6^ m) was introduced into a 10 mL sample vial. After complete evaporation of acetone, an aliquot of the BPGP aqueous solution (ranging from 0.015 to 1000 µg mL^−1^) was added into the sample vial. After 2 h incubation, the fluorescence spectra were captured and recorded via a fluorescence spectrophotometer.

The AKT inhibitor capivasertib ‐loaded branched poly(LAEMA^Cy5^)‐GFLG‐PTX was prepared using the solvent evaporation method. In brief, a methanol/acetonitrile mixed solution (0.4 mL) containing 1.125 mg of CAP was slowly added dropwise to a 5 mL aqueous solution of BPGP (2 mg mL^−1^, PTX:CAP = 1 (wt):1.5 (wt)) under stirring conditions. The mixture was stirred at room temperature until the organic solvents were completely evaporated. The remaining solution was then filtered through a membrane (0.45 µm) to remove unencapsulated CAP. Finally, the obtained filtrate was freeze‐dried to yield the desired product, BPGP@CAP. BPGP@CAP with different weight ratios of PTX and CAP was prepared by adjusting the weighting ratio of PTX and CAP. The loading contents of CAP in nanoassemblies were determined by HPLC system. The drug loading content (DLC) and the drug loading efficiency (DLE) were calculated using the following equations:

DLC (wt.%) = (weight of drug loaded/total weight of polymer and loaded drug) × 100

DLE (wt.%) = (weight of drug loaded/initial weight of drug) × 100

The hydrodynamic diameter of the obtained nanoassemblies were measured by dynamic light scattering (DLS, Brookhaven ZetaPALS, USA). The morphology of the obtained nanoassemblies was examined under a transmission electron microscope (TEM, Tecnai GF20S‐TWIN, USA). The enzyme‐responsive drug release capacity of nanoassembly was investigated under simulated physiological conditions, with and without the presence of papain.

### Cell Line and Animals

MFC cell, a cell line derived from GC, was acquired from the Chinese Academy of Medical Sciences (Shanghai, China), which were cultured in a temperature‐controlled incubator at 37 °C with 5% CO_2_ in a humidified environment. Male BALB/c nude mice (4‐6 weeks old, weighing 18–20 g) were procured from Hfk Bioscience Co., Ltd (Beijing, China). Subcutaneous GC MFC tumor models were generated through injecting 5 × 10^5^ MFC cells into the right back of the mice. Approval for all animal experiments was granted by the Animal Ethics Committee of West China Hospital, Sichuan University (Approval No. 2018154A).

### Clinical Tissue Sampling

The AKT expression and its relationship was examined with the prognosis of GC using the Gene Expression Omnibus (GEO) (https://www.ncbi.nlm.nih.gov/geo/) cohort data via Kaplan‐Meier Plotter (http://kmplot.com/analysis/index.php?p = background). Tumor and normal tissues of 100 GC patients were collected from the West China Hospital of Sichuan University. IHC was employed for assessing the expression of AKT. Based on the IHC scores, 100 GC patients were categorized into high and low AKT expression groups. The Kaplan–Meier method was used for the survival curve of both groups. This study regarding clinical tissue sampling received approval from the Ethics Committee of The West China Hospital of Sichuan University (2023 Review, No. 549) and all patients provided informed consent for participation in the study.

### Cellular Uptake, Endocytic Pathways, and Subcellular Localization

MFC cells were inoculated in a confocal chamber (1 × 10^5^ cells per well) and incubated overnight. They were then incubated with culture medium containing Cy5‐labeled BPGP@CAP (Cy5: 0.5 µg mL^−1^) for 1, 2, and 4 h, respectively. Following washing with PBS, Hoechst 33342 was added for nucleus staining. Cellular uptake of BPGP@CAP by MFC cells was determined by analyzing the Cy5 signal using laser scanning confocal microscopy (CLSM) (Nikon, Tokyo, Japan). The mean fluorescence intensity was obtained from semi‐quantitative analysis using Image J software. Additionally, flow cytometry (BD FACSCelesta, New Jersey, USA) was employed for detecting cellular uptake of BPGP@CAP by analyzing the Cy5 signal in MFC cells treated with Cy5‐labeled BPGP@CAP for various durations (1 h, 2 h, and 4 h).

To investigate the endocytic pathways of BPGP@CAP, MFC cells were pre‐treated with the culture medium containing 8 µg mL^−1^ chlorpromazine hydrochloride, 0.5 mm amiloride hydrochloride or 0.5 mm dynasore for 2 h. Subsequently, those MFC cells was incubated with medium containing Cy5‐labeled BPGP@CAP (Cy5: 0.5 µg mL^−1^) for an additional 2 h. Quantitative analysis of cellular uptake of BPGP@CAP was conducted using flow cytometry. Furthermore, to determine if the cellular uptake of BPGP@CAP was relied on energy, cells were exposed in the culture medium with Cy5‐labeled BPGP@CAP (Cy5: 0.5 µg mL^−1^) and incubated at 4 °C for a duration of 2 h and flow cytometry was employed to check the uptake level of BPGP@CAP.

For subcellular localization studies of BPGP@CAP, MFC cells were first exposed to Cy5‐labeled BPGP@CAP (Cy5: 0.5 µg mL^−1^) for 2 h. Following the staining of MFC cell lysosomes with Lyso Tracker Green and the nucleus with Hoechst 33342 for a duration of 15–20 min, observation and imaging of MFC cells were conducted using CLSM.

### Cell Cytotoxicity Assay

After MFC cells were inoculated in 96‐well plates (3 × 10^3^ cells per well) and grown overnight, MFC cells were exposed to various formulations (such as PTX, CAP, a mixture of PTX and CAP (PTX+CAP), BPGP, and BPGP@CAP) at different concentrations for 48 h. MFC cells treated with fresh culture medium were included as a control group. Following the treatment, those cells were exposed to a medium containing a 10% CCK‐8 solution and incubated at 37 °C for 1 h. A microplate reader (Tecan, Switzerland) was utilized to measure the OD450 value, which was then used to evaluate the relative cell viability.

### Cell Apoptosis and Cell Cycle Assay

MFC cells were inoculated in a 6‐well plate with 2 × 10^5^ cells per well and grown for 12 h. Subsequently, those cells were incubated with the culture medium containing PTX, CAP, PTX + CAP, BPGP, or BPGP@CAP (the PTX concentration: 2.0 µg mL^−1^ and the corresponding concentration of CAP: 2.1 µg mL^−1^) for 48 h. MFC cells treated with fresh culture medium were included as a control group. Those cells were collected and stained with an Annexin V‐PI reagent for 30 min. Flow cytometry was employed for assessing apoptosis of MFC cells.

For cell cycle assays, cells that received treatment with PTX, CAP, PTX + CAP, BPGP, or BPGP@CAP (the PTX concentration: 2.0 µg mL^−1^ and the corresponding concentration of CAP: 2.1 µg mL^−1^) for a duration of 48 h were collected and subjected to overnight fixation in 75% ethanol. Following gentle washing with PBS, they were delicately incubated with a 0.5 mL solution of the PI/RNase dye. The flow cytometry was used to discern the cellular distribution at different cell cycle phases. Each experiment had three replicates, and the acquired data was analyzed through the Flowjo software.

### Morphological Examination of Microtubules and Microfilaments

The culture medium containing PTX, CAP, PTX + CAP, BPGP, or BPGP@CAP was used to treat MFC cells for 24 h to examine the morphology of microtubules. Following washing with PBS, those MFC cells were fixed with a 4% paraformaldehyde solution. After washing with PBS containing 0.1% Triton X‐100, the cells were subjected to Tubulin‐tracker Red staining for 1 h. Subsequently, nucleus staining was performed with DAPI. The cells were then observed and imaged under CLSM. For morphological analysis of microfilaments, MFC cells were stained with Actin‐tracker Green and DAPI and they were then observed and imaged via CLSM.

### Transcriptome Analysis by RNA‐Seq

MFC cells were inoculated in a 6‐well plate (2 × 10^5^ cells per well) and grown for 12 h. Thereafter, they were exposed to BPGP@CAP (the PTX concentration: 2.0 µg mL^−1^ and the corresponding concentration of CAP: 2.1 µg mL^−1^) for a duration of 48 h. MFC cells treated with fresh culture medium were included as a control group. Total RNA of MFC cells treated with BPGP@CAP in fresh culture medium was extracted. Genechem Co., Ltd (Shanghai, China) conducted the subsequent steps of quality control, library preparation, and RNA sequencing. Gene Ontology (GO) and KEGG pathway analyses were performed through the R package. Significant differences between the BPGP@CAP treatment group and the control cohort were robustly identified with a *p*‐adjust < 0.05 and a |log2FC| > 1.

### Western Blots

MFC cells were treated with PTX, CAP, PTX + CAP, BPGP, or BPGP@CAP (the PTX concentration: 2.0 µg mL^−1^ and the corresponding concentration of CAP: 2.1 µg mL^−1^) for 48 h. Untreated MFC cells were set as a control group. After collecting the cells from each experimental group, total proteins were collected from cell lysates in a lysis buffer. Subsequently, they underwent SDS‐PAGE gel electrophoresis and were then transferred to a 0.45 µm PVDF membrane (Millipore). Following a blockade using a 5% milk TBST solution, the membrane was subjected to an overnight incubation with primary antibodies in a refrigerator at 4°C. After a thorough TBST wash, HRP‐conjugated secondary antibodies were applied to the membrane and incubated at room temperature for 1 h. Detection of the proteins was achieved through the Syngene GeneGenius gel imaging system (Syngene, Cambridge, UK).

### In Vivo Pharmacokinetics Studies

Normal male balb/c nude mice were randomly allocated into two groups (n = 5) for intravenous administration of free Cy5 and Cy5‐labelled BPGP@CAP (Cy5: 1.5 mg kg^−1^). At various time points (0 min, 5 min, 15 min, 30 min, 1 h, 2 h, 4 h, 6 h, 8 h, 12 h, 16 h, and 24 h) after injection, blood samples were obtained from the orbital vein and immediately mixed with double distilled water. After the samples were then supplemented with DMSO and refrigerated overnight at 4 °C, they were centrifuged at 12 000 rpm for 15 min, and the supernatant from each sample was transferred to a 96‐well plate for fluorescence measurement using a microplate reader. The pharmacokinetic parameters were calculated using the PKsolver software.

### In Vivo Distribution

The MFC tumor‐bearing nude mice (*n* = 3) received intravenous injections via the tail with free Cy5 and Cy5‐labeled BPGP@CAP (Cy5: 1.5 mg kg^−1^) in order to assess their in vivo biodistribution. At various time points following the injection (1 h, 2 h, 3 h, 6 h, 9 h, 12 h, and 24 h), the mice were observed and imaged using an IVIS Spectrum (Caliper Life Sciences, Cambridge, UK). Upon completion of the imaging experiment, tumor samples from the mice were obtained and subjected to frozen sectioning. After staining with DAPI to label cell nuclei, the sections were observed and imaged using CLSM.

### In Vivo Anti‐Tumor Study

The mice with MFC tumors received different formulations via tail vein injection (*n* = 5): (1) Saline; (2) PTX; (3) CAP; (4) PTX + CAP; (5) BPGP; and (6) BPGP@CAP (the PTX concentration: 8.0 mg kg^−1^ and the corresponding concentration of CAP: 8.5 mg kg^−1^). The drugs were administered every other day until the completion of the treatment. The body weight of the mice, the length (L) and width (W) of the tumor were regularly recorded. The tumor volume (V) was determined using the formula: V = (L × W^2^)/2.

After the treatment, the mice were humanely euthanized by cervical dislocation, and their tumors were surgically excised and weighed. Furthermore, the tumors and vital organs underwent a process of fixation, embedding, and sectioning. The tumor sections were subjected to staining with Ki67 and TUNEL to evaluate proliferation and apoptosis of tumor cells. In addition, hematoxylin and eosin (H&E) staining was performed to assess the biocompatibility of these formulations.

Moreover, the biosafety of these formulations was evaluated in MFC tumor‐bearing nude mice. Hematological and biochemical analyses were conducted using collected whole blood and serum samples. Healthy balb/c nude mice were included as a control group for comparison.

### Statistical Analysis

GraphPad Prism 9 software was employed to analyze data obtained from at least three independent measurements. The results were presented as the mean ± standard deviation (± S.D.). Statistical significance was determined by performing a two‐tailed t‐test between two groups, and a *P*‐value less than 0.05 was considered to be statistically significant.

## Conflict of Interest

The authors declare no conflict of interest.

## Supporting information

Supporting Information

## Data Availability

The data that support the findings of this study are available from the corresponding author upon reasonable request.
