# Supplementary material for: Enzyme‐Responsive Branched Glycopolymer‐Based Nanoassembly for Co‐Delivery of Paclitaxel and Akt Inhibitor toward Synergistic Therapy of Gastric Cancer
Source: Adv Sci (Weinh). 2023 Nov 12;11(2):2306230. doi: 10.1002/advs.202306230 (PMC10787093; doi:10.1002/advs.202306230)
Supplement: Supplementary file 1 — Supporting Information [file ADVS-11-2306230-s001.pdf]

ADVANCED SCIENCE
Open Access

Supporting Information

for *Adv. Sci.*, DOI 10.1002/advs.202306230

Enzyme-Responsive Branched Glycopolymer-Based Nanoassembly for Co-Delivery of Paclitaxel and Akt Inhibitor toward Synergistic Therapy of Gastric Cancer

Xiaohai Song, Hao Cai, Zhaochen Shi, Zhiqian Li, Xiuli Zheng, Kun Yang, Qiyong Gong, Zhongwei Gu, Jiankun Hu and Kui Luo**

Supporting Information

Enzyme-Responsive Branched Glycopolymer-Based Nanoassembly for Co-Delivery of Paclitaxel and AKT Inhibitor towards Synergistic Therapy of Gastric Cancer

Xiaohai Song, Hao Cai, Zhaochen Shi, Zhiqian Li, Xiuli Zheng, Kun Yang, Qiyong Gong, Zhongwei Gu, Jiankun Hu [*], and Kui Luo [*]

X. Song, Z. Li, X. Zheng, K.Yang, Q. Gong, Z. Gu, J. Hu, K. Luo
Department of General Surgery, Gastric Cancer Center, Department of Radiology, Huaxi MR Research Center (HMRRC), Frontiers Science Center for Disease-Related Molecular Network, Laboratory of Gastric Cancer, State Key Laboratory of Biotherapy, West China Hospital, Sichuan University, Chengdu 610041, China.
*E-mail: hujiankun@scu.edu.cn (Jiankun Hu), luokui@scu.edu.cn (Kui Luo)
H. Cai
Department of Thoracic Surgery and Institute of Thoracic Oncology, Frontiers Science Center for Disease-related Molecular Network, West China Hospital of Sichuan University, Chengdu, 610097, China.
Z. Shi
West China School of Medicine, Sichuan University, Chengdu, 610041, China.
Q. Gong, K. Luo
Functional and Molecular Imaging Key Laboratory of Sichuan Province, West China Hospital, Sichuan University, and Research Unit of Psychoradiology, Chinese Academy of Medical Sciences, Chengdu 610041, China
Q. Gong,
Department of Radiology, West China Xiamen Hospital of Sichuan University, Xiamen 361000, China
Z. Gu
Research Institute for Biomaterials, Tech Institute for Advanced Materials, College of Materials Science and Engineering, NJTech-BARTY Joint Research Center for Innovative Medical Technology, Suqian Advanced Materials Industry Technology Innovation Center, Jiangsu Collaborative Innovation Center for Advanced Inorganic Function Composites, Nanjing Tech University, Nanjing 211816, China

Supplement information materials

Characterization methods

Characterizations of monomers and polymer conjugates were conducted by ^{1}H NMR (400 MHz Bruker Avance II NMR spectrometer, Germany) for their structure confirmation. The weight-average (M_w) and number-average (M_n) molecular weights of polymers were determined by Gel Permeation Chromatography (GPC) with a GPC column of Shodex Asahipak GF-510 HQ (7.5 mm ID × 300 mmL). The injection volume was 50 μL of 2 mg mL^{-1} sample solutions. The mobile phase was a 0.2 M lithium chloride sodium solution (H_2O: DMF = 35:65, v/v) at a flow rate of 0.5 mL/min (45 °C). M_w, M_n and polydispersity index (PDI) were measured using Pullulan as a standard. High performance liquid chromatography (HPLC) analyses were performed on a Shimadzu prominence HPLC system.

Synthesis of functionalized compounds

2-lactobionamidoethyl methacrylamide (LAEMA),[1] *N*-[2-(pyridin-2-yldisulfanyl) ethyl] methacrylamide (MA-PySS)[2] and (3-(2,5-dioxo-2,5-dihydro-1H-pyrrol-1-yl)propanoyl)glycylphenylalanylleucylglycine (Maleimide-GFLG-OH)[3] were synthesized according to the literature. Cross linkers, MA-GFLG-MA and Maleimide-GFLG-PTX, were synthesized with details in each step shown in Scheme S1.

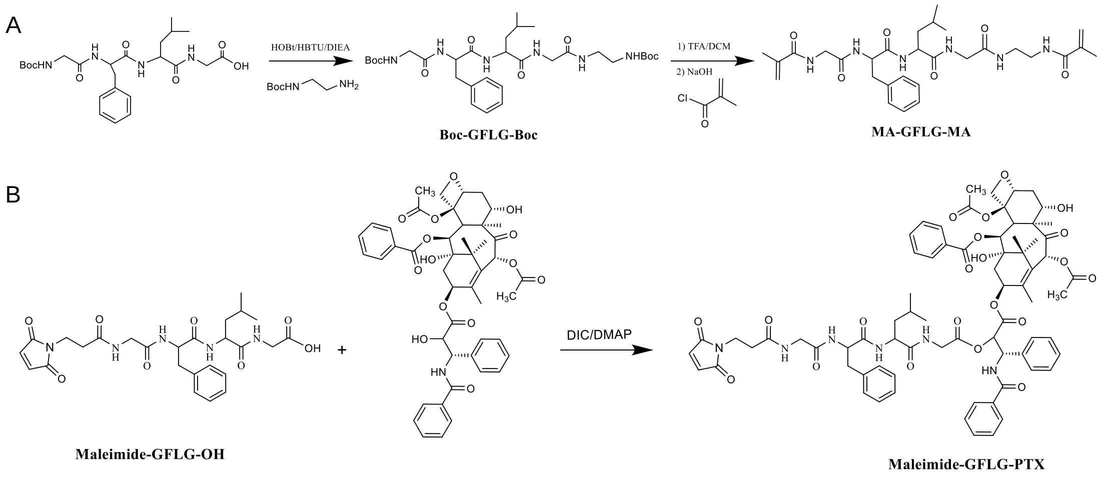

Scheme S1. Synthesis route of MA-GFLG-MA and Maleimide-GFLG-PTX.

Synthesis of MA-GFLG-MA

The Boc-GFLG-Boc compound was synthesized following a previously reported method.[4] Subsequently, Boc-GFLG-Boc (690 mg, 1.09 mmol) was dissolved in 9 mL of dichloromethane (DCM) in an ice bath, followed by gradual addition of 9 mL of trifluoroacetic acid. The reaction mixture was mixed at room temperature for 5 h. Afterward, the solvent was removed by rotary evaporation, and the remaining residue was washed twice with diethyl ether. The solid residual was vacuum-dried, yielding a white solid product.

The deprotection products of Boc-GFLG-Boc (400 mg, 0.63 mmol) was dissolved in a mixed solution of 6 mL acetonitrile (ACN) and water (1:4, v/v) under an ice bath. A trace amount of 4-methoxyphenol was added to the reaction mixture, followed by gradual addition of an ACN solution containing methylacrylyl chloride (MA-Cl) (0.183 mL, 1.89 mmol). Concentrated sodium hydroxide (NaOH) was used during the reaction to maintain the pH of the reaction mixture at 8 to 9. After 1 h of reaction at 0° C, the reaction proceeded for an additional hour at room temperature. Upon completion, the organic solvent was removed by rotary evaporation. The remaining residue was diluted with a large amount of ethyl acetate. Dilute hydrochloric acid was added to adjust the pH of the reaction mixture to be 2 to 3. The organic layer was collected, dried with anhydrous sodium sulfate, and concentrated to approximately 10 mL. The solution was left at 4 °C for crystallization. Subsequently, the product was collected by filtration, yielding a white solid product of 0.34 g. ^{1}H NMR (400 MHz, DMSO-*d6*, δ): 8.28-7.72 (m, 6H), 7.36-7.06 (m, 5H), 5.68 (d, J = 13.7 Hz, 2H), 5.34 (d, J = 17.5 Hz, 2H), 4.53 (td, J = 8.7, 4.3 Hz, 1H), 4.25 (q, J = 7.5 Hz, 1H), 3.90-3.50 (m, 4H), 3.16 (s, 4H), 3.03 (dd, J = 13.9, 4.3 Hz, 1H), 2.79 (dd, J = 13.7, 9.3 Hz, 1H), 1.64-1.46 (m, 3H), 0.86 (dd, J = 18.9, 6.4 Hz, 6H). LC-MS (ES^+): 571.3 $[M+H]^+$.

Synthesis of Maleimide-GFLG-PTX

Maleimide-GFLG-OH (500 mg, 0.92 mmol), PTX (943 mg, 1.1 mmol), and DMAP (7.1 mg, 0.06 mmol) were accurately weighed and placed into a round-bottom flask. Under a nitrogen atmosphere, 30 mL of DCM was added to ensure complete dissolution of the reactants. With continuous stirring over an ice bath, a solution of DIC (285 μL, 1.84 mmol) in DCM was slowly dripped into the reaction mixture. Subsequently, the reaction mixture was kept at 4 °C for 16 h. After removal of the solvent under a reduced pressure, the resulting residue was dissolved in 200 mL of DCM. The mixture was subjected to sequential washing with a saturated sodium bicarbonate solution, dilute hydrochloric acid, and a saturated sodium chloride solution. Each washing step was performed three times. Collect the organic layer, dry it with anhydrous sodium sulfate, and remove the solvent through vacuum distillation. The crude product was subjected to column purification, yielding a white solid product of 523 mg. ^{1}H NMR (400 MHz, DMSO-*d6*, δ): 9.25 (t, J = 8.0 Hz, 1H), 8.34-7.81 (m, 8H), 7.79-7.63 (m, 3H), 7.62-7.38 (m, 7H), 7.30-7.13 (m, 6H), 6.99 (s, 2H), 6.30 (d, J = 5.8 Hz, 1H), 5.84 (t, J = 8.9 Hz, 1H), 5.52 (dd, J = 16.9, 8.3 Hz, 1H), 5.41 (q, J = 8.8 Hz, 2H), 4.98-4.83 (m, 2H), 4.63 (d, J = 14.5 Hz, 1H), 4.53 (td, J = 8.8, 4.5 Hz, 1H), 4.35 (dt, J = 15.5, 8.3 Hz, 1H), 4.16-3.90 (m, 6H), 3.67-3.52 (m, 4H), 3.11-2.98 (m, 1H), 2.87-2.70 (m, 1H), 2.43-2.27 (m, 3H), 2.25-

2.18 (m, 3H), 2.10 (s, 3H), 1.87-1.71 (m, 4H), 1.69-1.54 (m, 2H), 1.53-1.38 (m, 6H), 1.01 (d, J = 10.3 Hz, 6H), 0.90-0.77 (m, 6H).

Synthesis of LAEMA-based branched polymeric prodrug

A cathepsin B-sensitive branched polymer prodrug (branched poly(PLAEMA)-GFLG-PTX, BPGP) was synthesized through a combination of RAFT polymerization and thiol-ene reaction. First, a high-molecular-weight chain transfer agent based on LAEMA was prepared. Subsequently, this agent was copolymerized with MA-GFLG-MA, a GFLG-functionalized crosslinker, and MA-PySS, resulting in the formation of a branched polymer scaffold. After deprotection and exposure of thiol groups on the branched polymer scaffold, the maleimide-modified GFLG-functionalized PTX prodrug was covalently coupled to the polymer via thiol-ene reaction, yielding the target product. The synthetic route is illustrated in the Scheme S2.

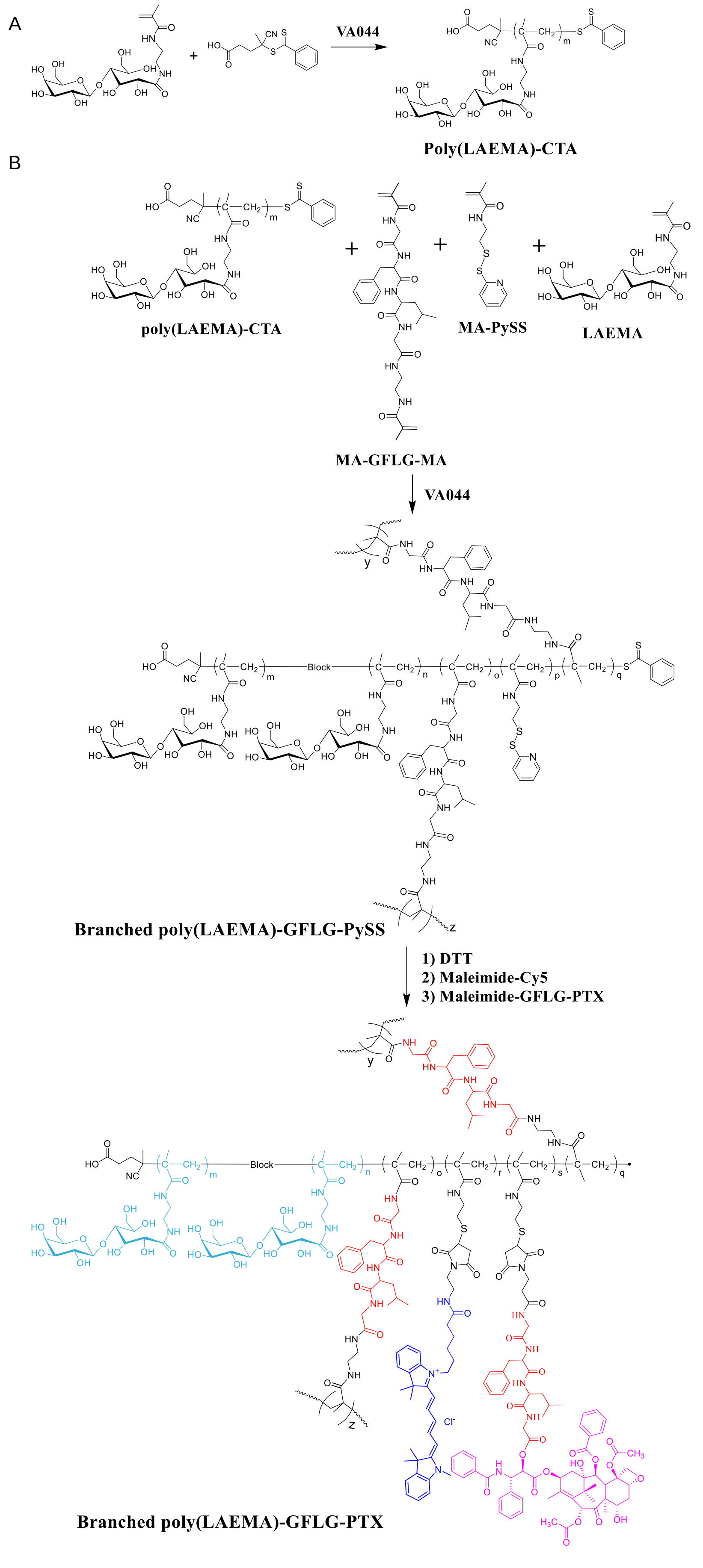

Scheme S2. Synthesis route of branched poly(LAEMA)-GFLG-PTX.

IHC and score

Paraffin-embedded clinical tissue sections were subjected to antigen retrieval; endogenous peroxidase was blocked using 3% hydrogen peroxide; and BSA was evenly applied to cover the tissue sections and incubated at room temperature. A primary antibody was applied on the tissue sections for overnight incubation at 4°C in a humidified chamber. After washing with PBS, the tissues were incubated with an HRP-conjugated secondary antibody at room temperature. After washing with PBS, DAB was used for color development, and the reaction was terminated by rinsing with tap water. Counterstaining was performed with hematoxylin, followed by dehydration and slide sealing. Image acquisition and scoring analysis were conducted.

The immunohistochemical scoring was determined by multiplying the percentage of positive cells and the staining intensity. The scoring system for the percentage of positive cells was set as follows: 0 score for less than 5% of positive cells; 1 score for greater than 5% but less than 25% of positive cells; 2 score for greater than 25% but less than 50% of positive cells; 3 score for greater than 50% but less than 75% of positive cells; and 4 score for greater than 75% of positive cells. The staining intensity was scored as 0 for negative (no color), 1 for weak positive (light yellow), 2 for moderate positive (light brown), and 3 for strong positive (brown).[5] Table S3 lists the scoring criteria for IHC.

After incorporating the immunohistochemical scoring and survival rates into the X-tile software,[6] we calculated the score that maximizes the survival difference and designated it as the cut-off value. Basing on the score, we classified the patients into an AKT low-expression group and an AKT high-expression group.

Tumor regression grading (TRG)

The TRG was evaluated by two experienced pathologists independently according to the CAP TRG classification.[7] TRG was classified as follows: Grade 0 (complete response) for the absence of viable residual cancer cells; Grade 1 (moderate response) for a small number of residual cancer cells; Grade 2 (minimal response) for residual tumors outgrown by fibrosis; and Grade 3 (poor response) for abundant residual cancer.

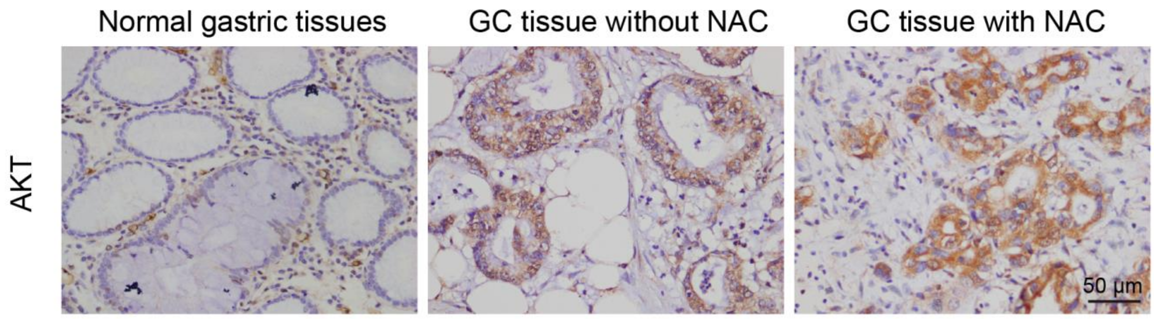

Figure S1. IHC for AKT expression in normal gastric tissues (GC), GC samples with and without neoadjuvant chemotherapy (NAC). Scale bar = 50 μm.

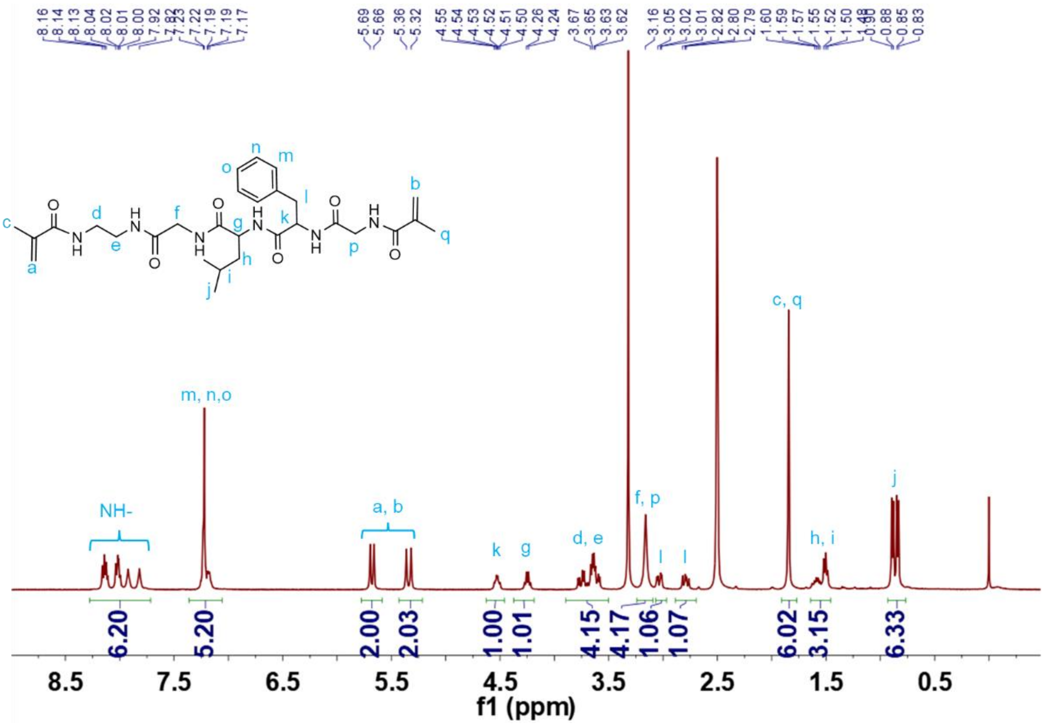

Figure S2. ^{1}H NMR spectrum of MA-GFLG-MA in DMSO-d_6.

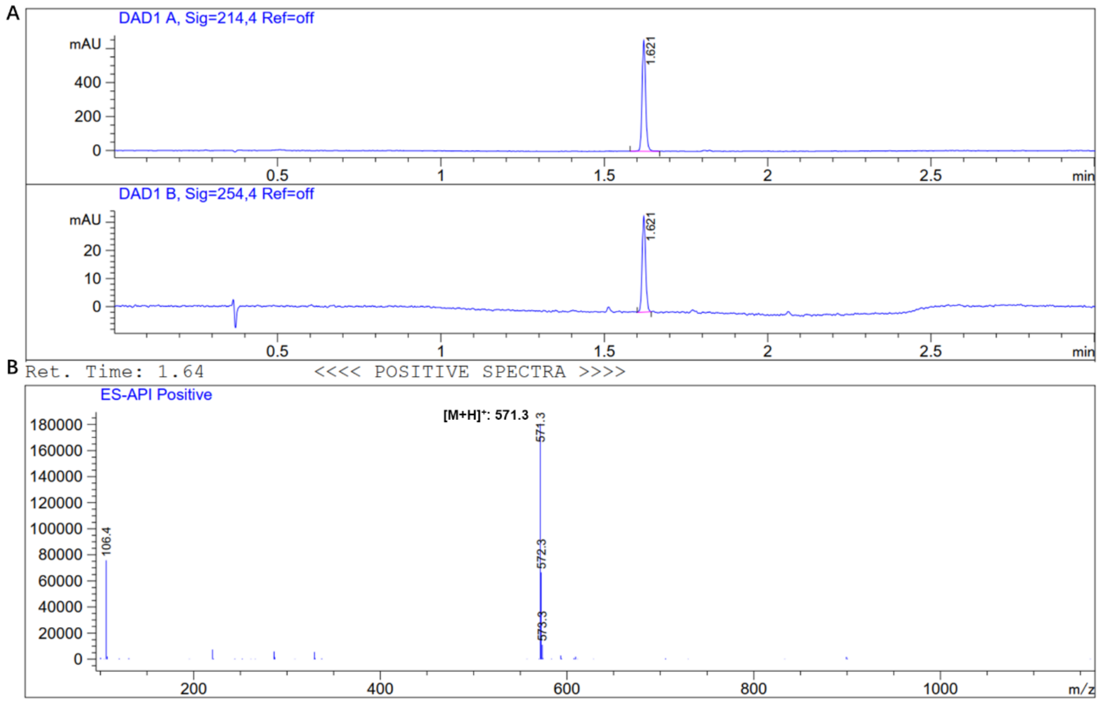

Figure S3. LC-MS spectrum of MA-GFLG-MA. The product peak at 1.621 min (A), and a single ion peak at 571.3 $[M+H]^+$ (B).

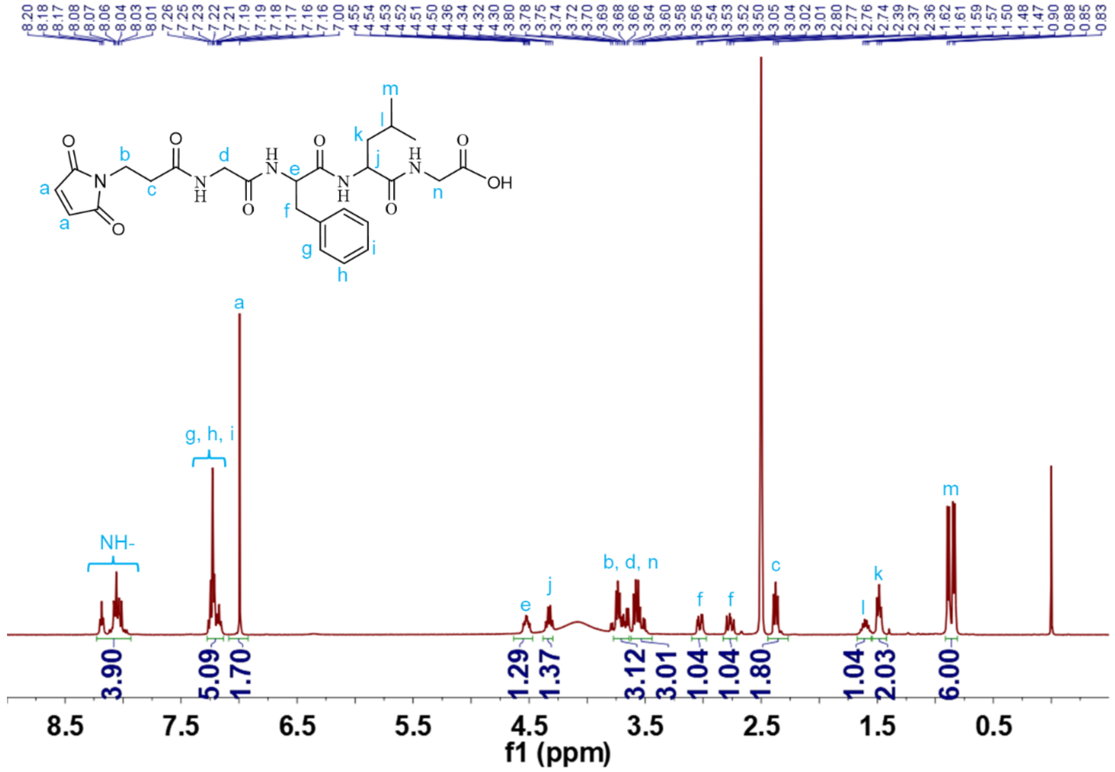

Figure S4. ^{1}H NMR spectrum of maleimide-GFLG-OH in DMSO-d_6.

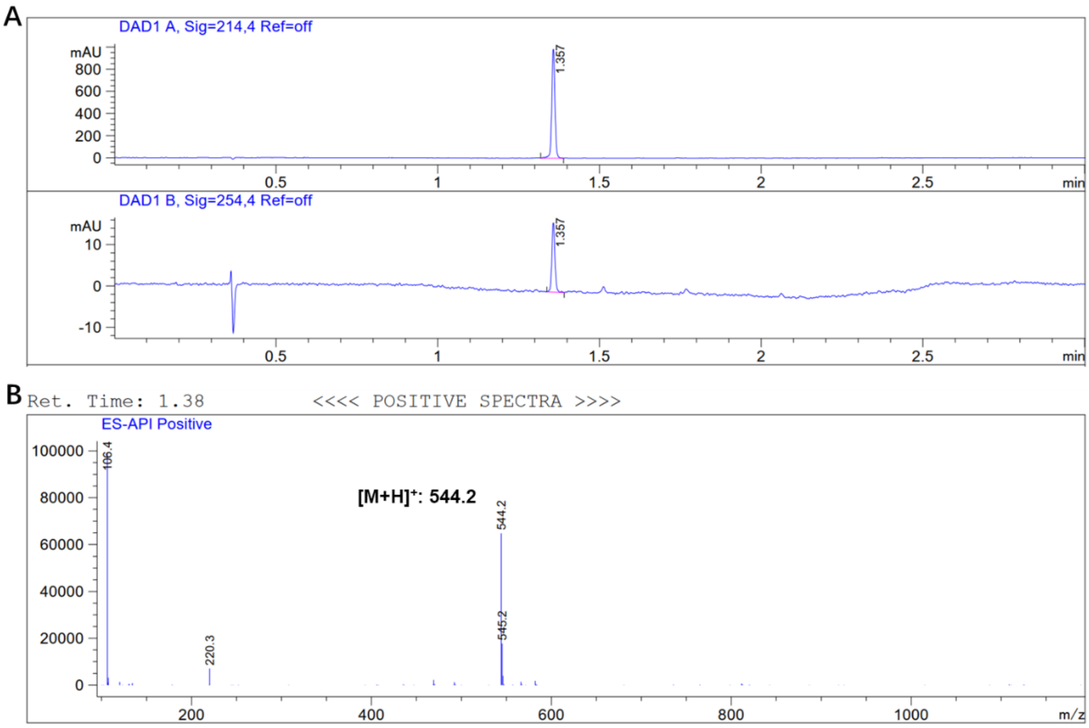

Figure S5. LC-MS spectrum of maleimide-GFLG-OH. The product peak at 1.357 min (A), and a single ion peak at 544.2 $[M+H]^+$ (B).

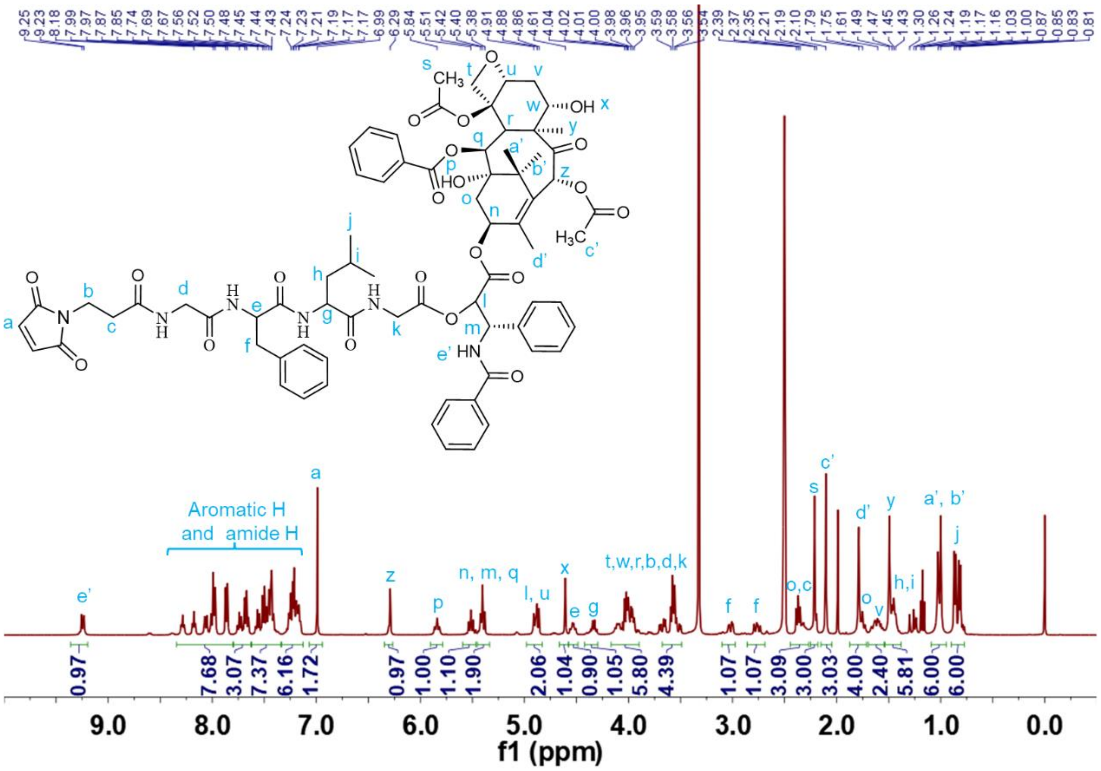

Figure S6. ^{1}H NMR spectrum of maleimide-GFLG-PTX in DMSO-d_6.

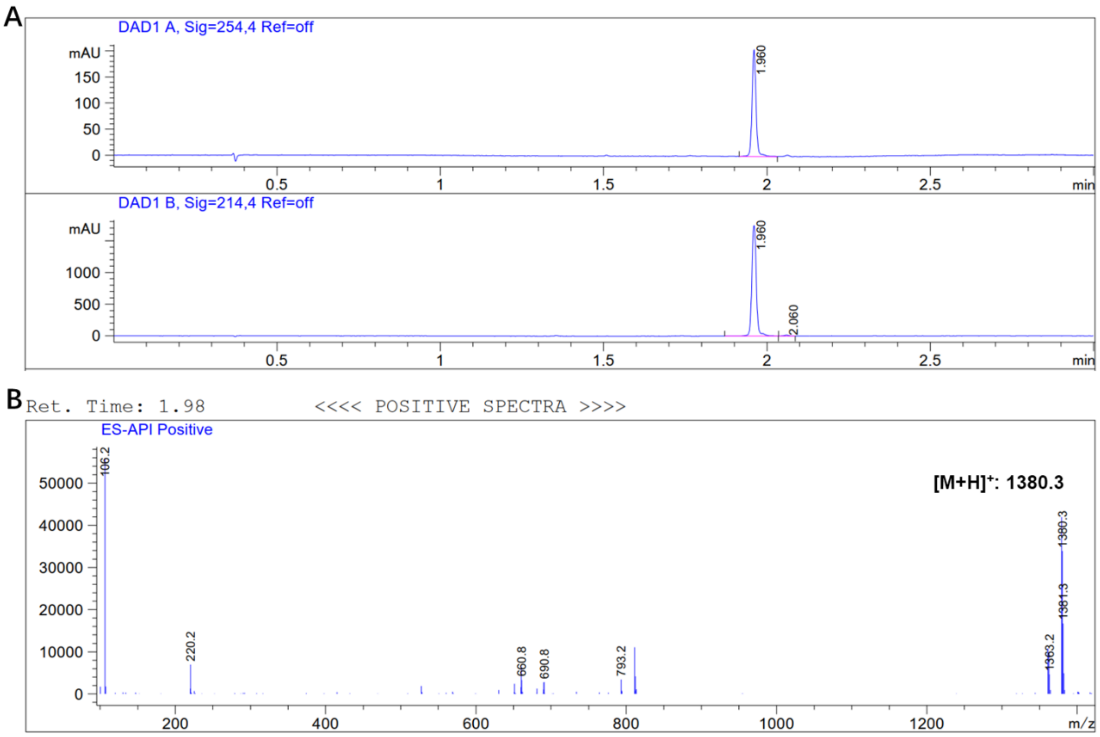

Figure S7. LC-MS spectrum of Maleimide-GFLG-PTX. The product peak at 1.960 min (A), and a single ion peak at 1380.3 $[M+H]^+$ (B).

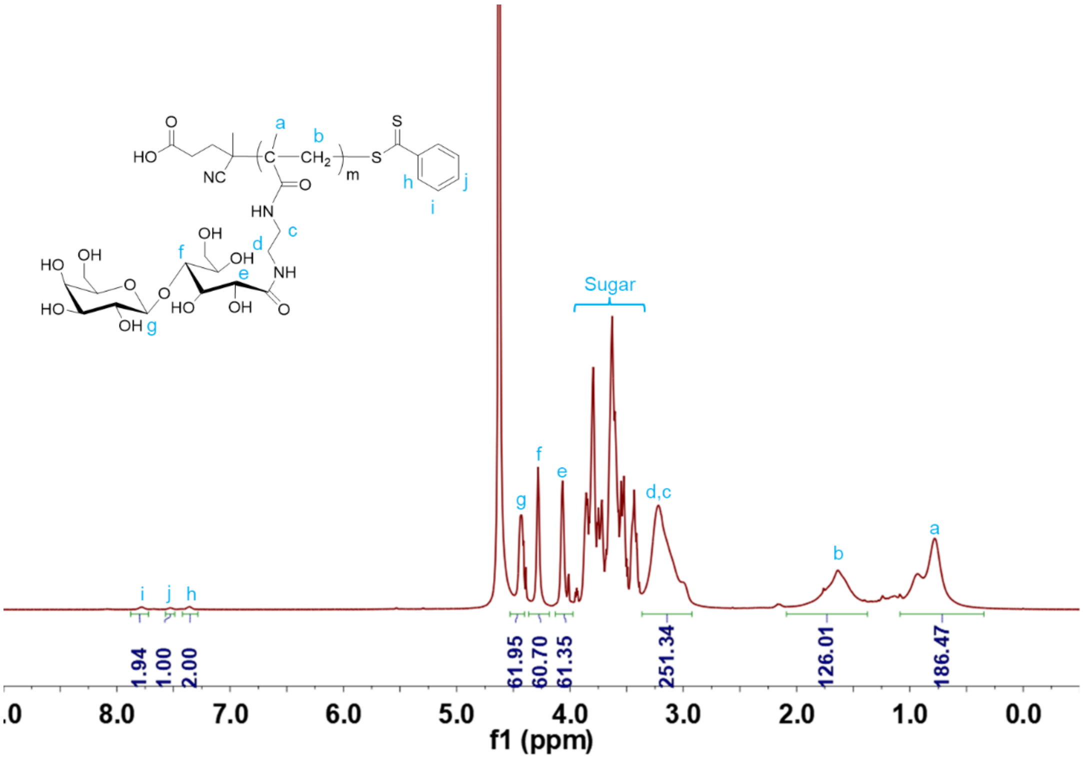

Figure S8. ^{1}H NMR spectrum of poly (LAEMA)-CTA in D_2O.

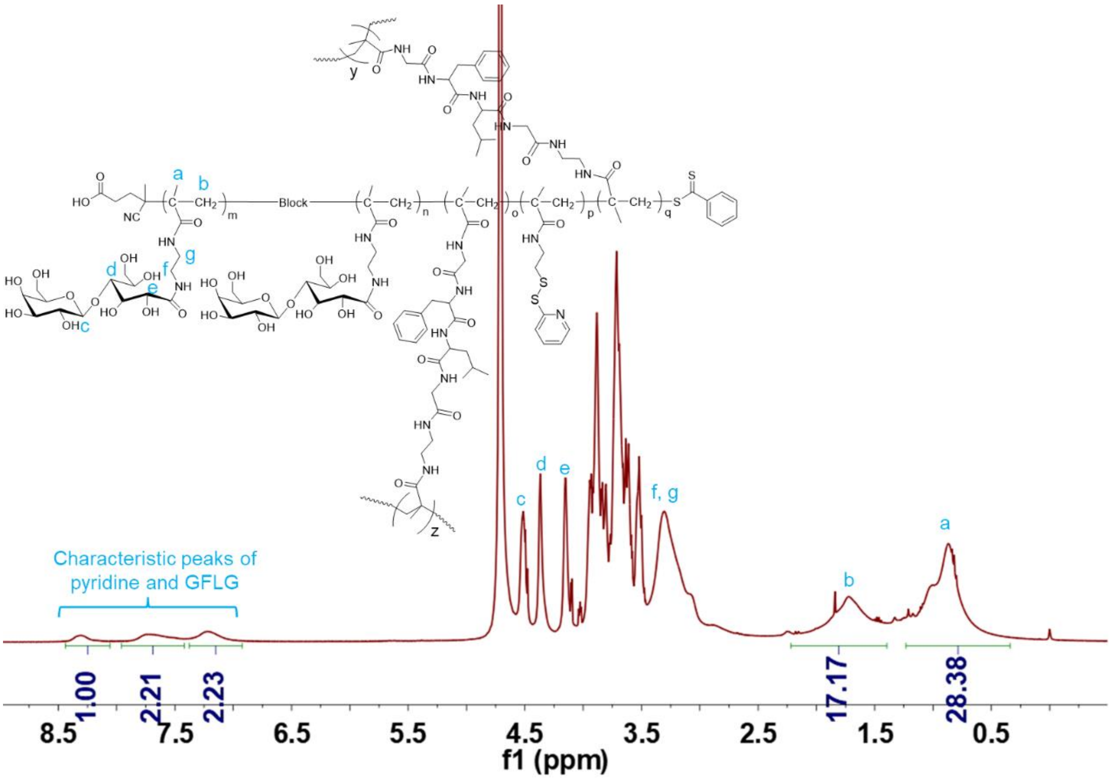

Figure S9. ^{1}H NMR spectrum of branched poly(LAEMA)-GFLG-PySS in D_2O.

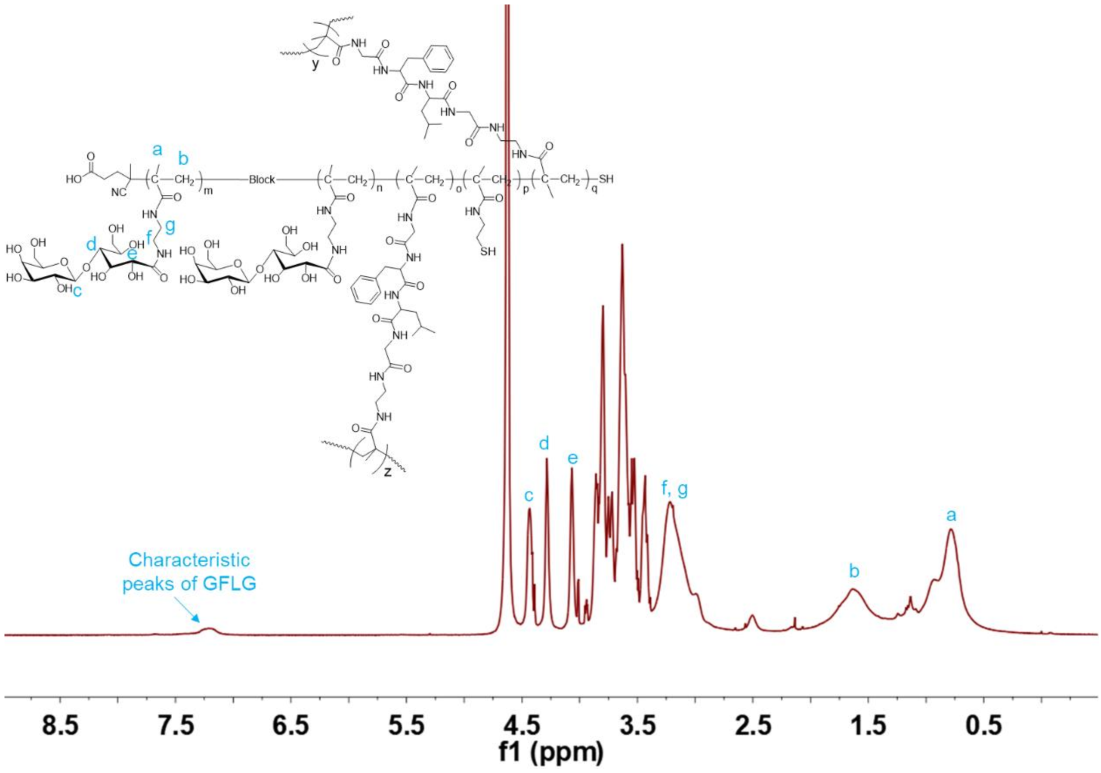

Figure S10. ^{1}H NMR spectrum of branched poly(LAEMA-co-GFLG-SH) in D_2O.

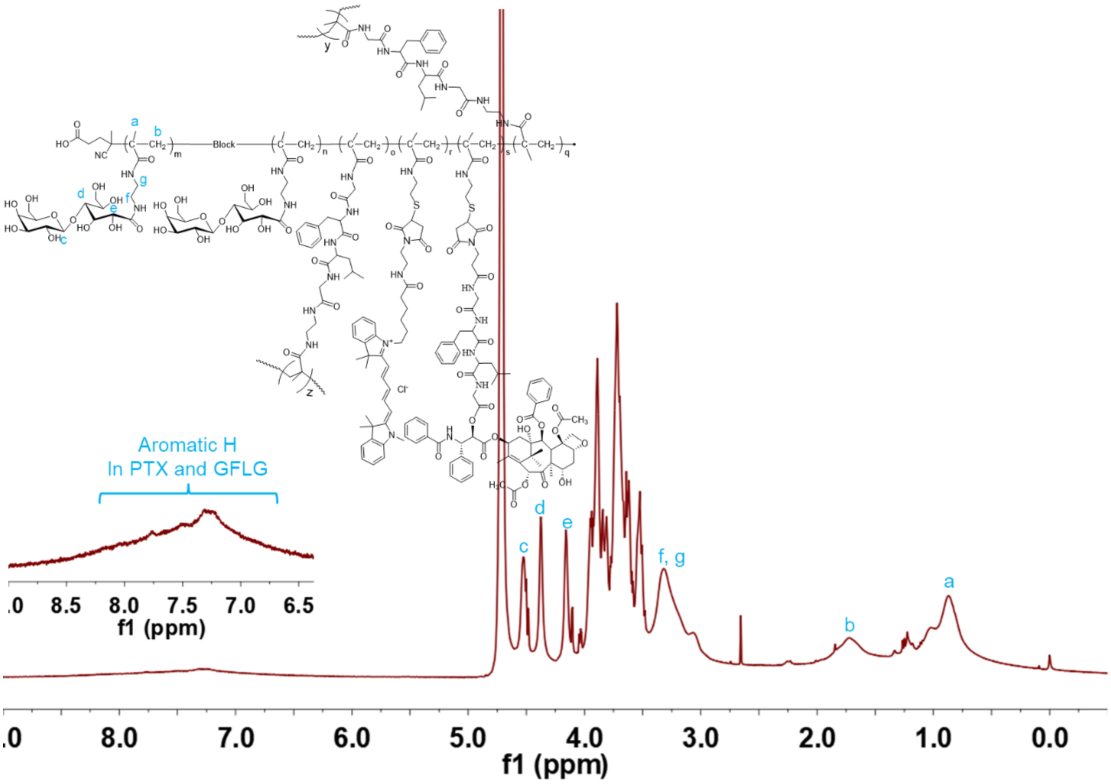

Figure S11. ^{1}H NMR spectrum of branched poly(LAEMA^{Cy5}-co-GFLG-PTX) in D_2O.

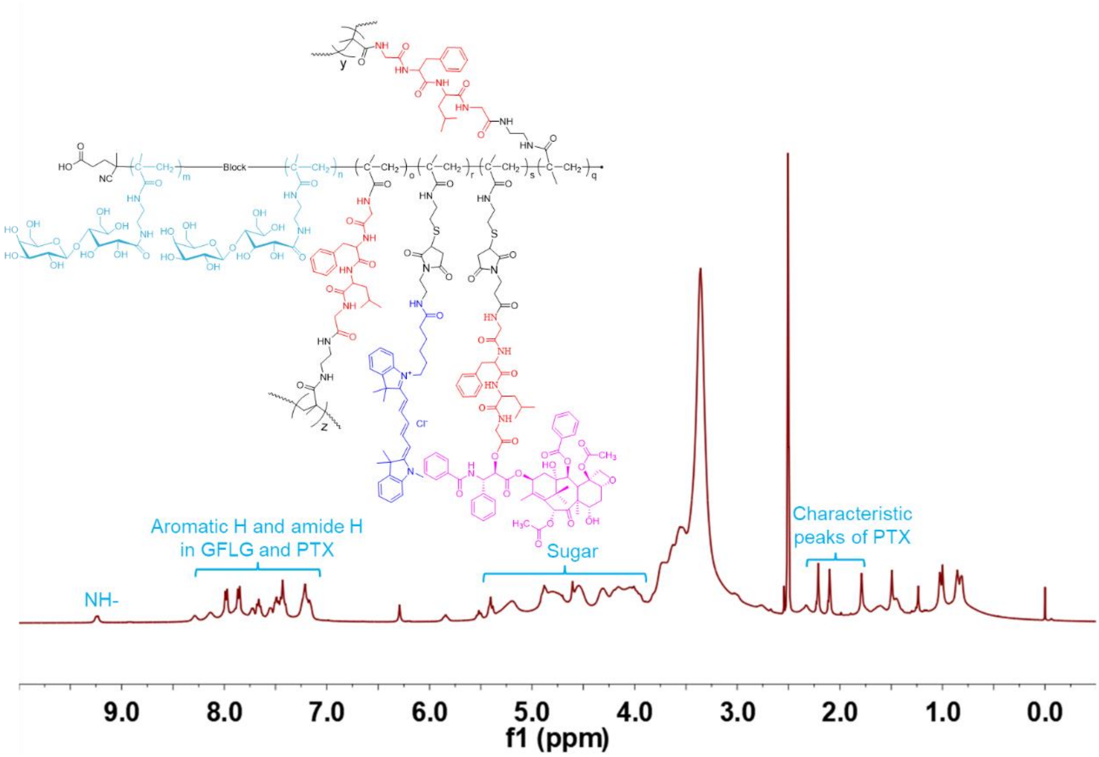

Figure S12. ^{1}H NMR spectrum of branched poly(LAEMA^{Cy5}-co-GFLG-PTX) in DMSO.

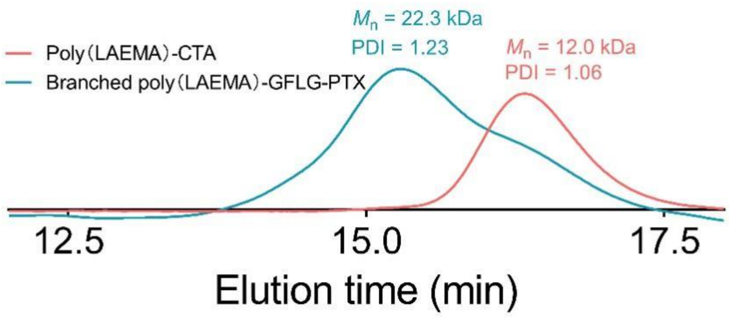

Figure S13. GPC chromatogram of poly (LAEMA)-CTA and branched poly(LAEMA)-GFLG-PTX.

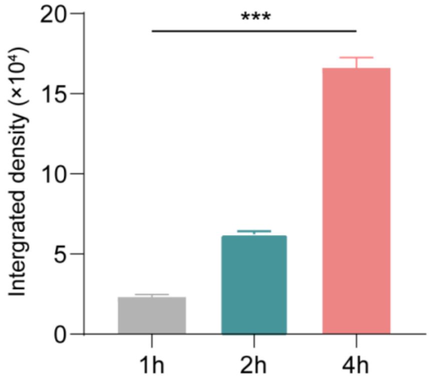

Figure S14. Semi-quantitative analysis of mean fluorescence intensity (MFI) of Cy5 in MFC cells after exposure to Cy5-labelled BPGP@CAP for 1 h, 2 h and 4 h via Image J (n = 3). Data were expressed as mean ± SD. The statistical significance was displayed by two-sided unpaired Student's t-test, ****p* < 0.001.

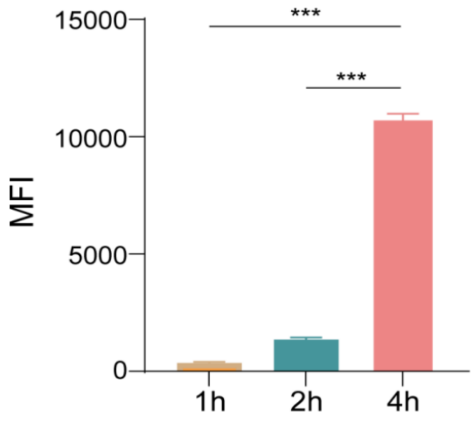

Figure S15. Semi-quantitative analysis of MFI in MFC cells after exposure to Cy5-labelled BPGP@CAP for 1 h, 2 h and 4 h (n = 3). Data were presented as mean ± SD. Statistical significance was determined using a two-sided unpaired Student's t-test, ***p < 0.001.

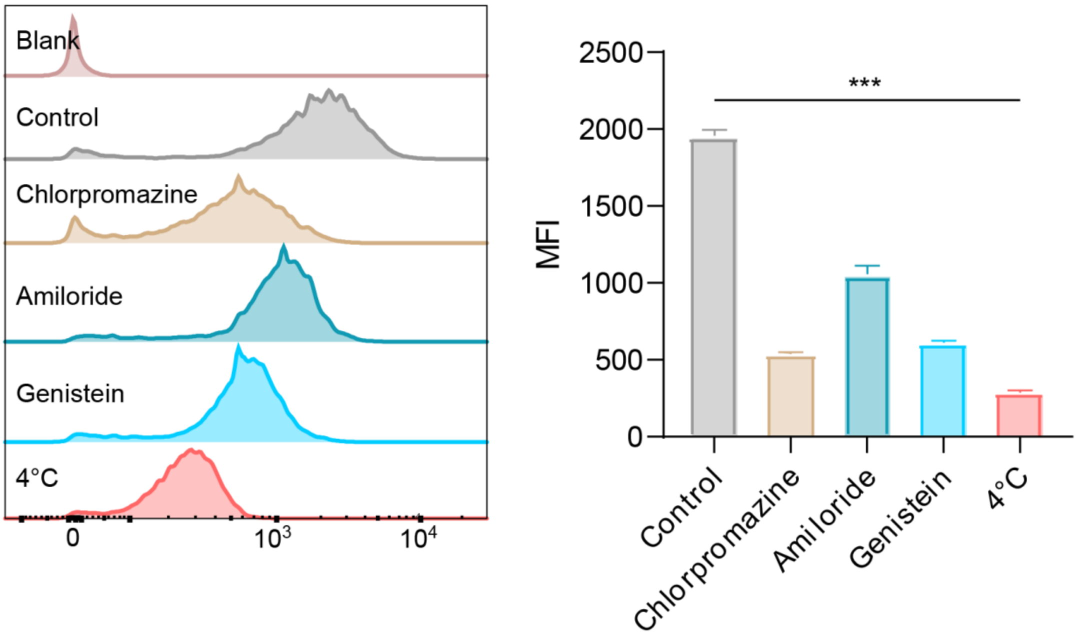

Figure S16. Flow cytometry diagram and MFI of Cy5-labelled BPGP@CAP uptaken by MFC cells after exposure to different inhibitors or at a low temperature (n=3). Data were shown as mean ± SD. The statistical significance was displayed by two-sided unpaired Student's t-test, ***$p < 0.001$.

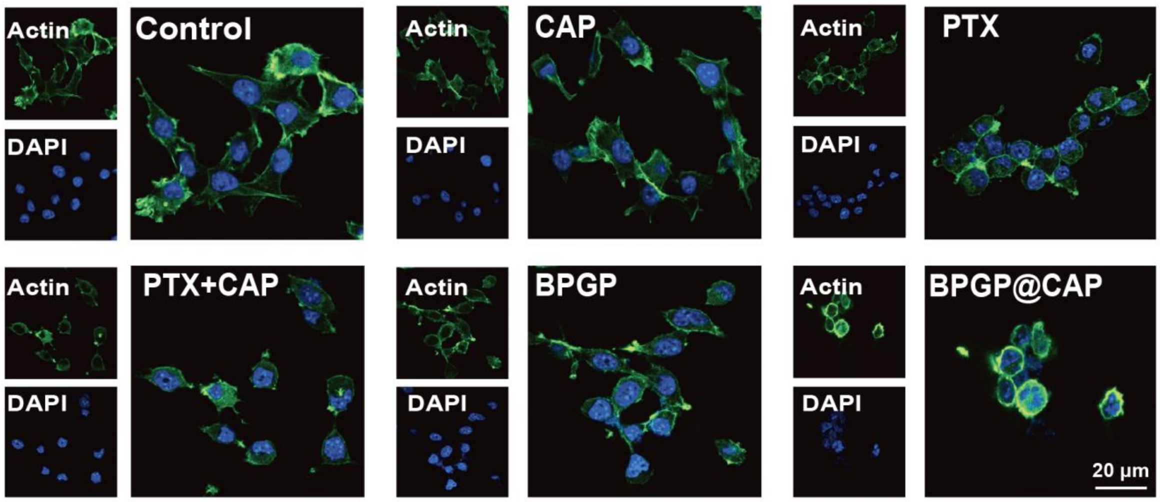

Figure S17. Microfilament changes in MFC cells induced by various treatments for 24 h. Green for Actin-Tracker Green and blue for cell nuclei stained by DAPI. Scale bar = 20 µm.

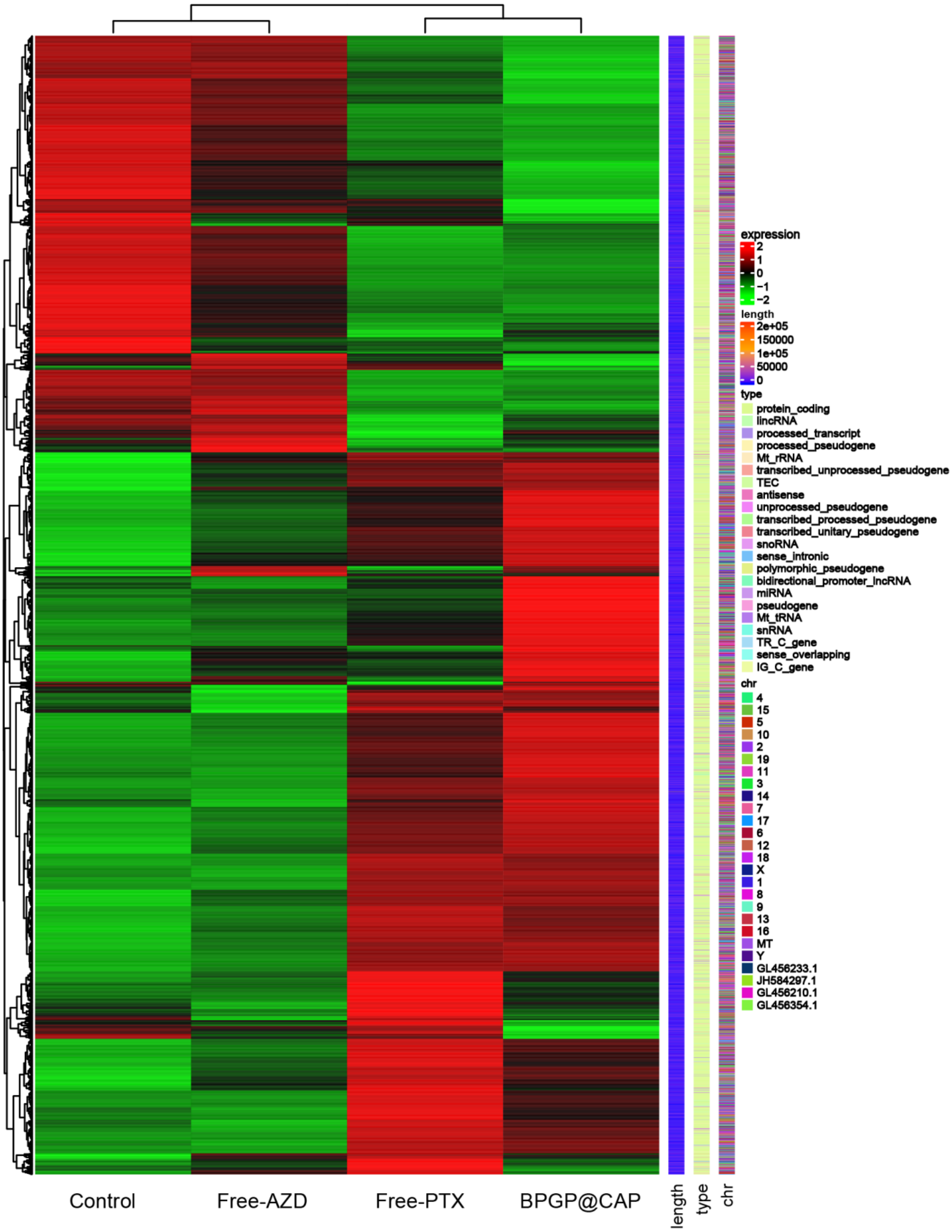

Figure S18. Heatmap of differentially expressed genes (DEGs) in MFC cells after different treatments measured by RNA-seq. Red for upregulation and green for downregulation. The P adjusted value was < 0.05.

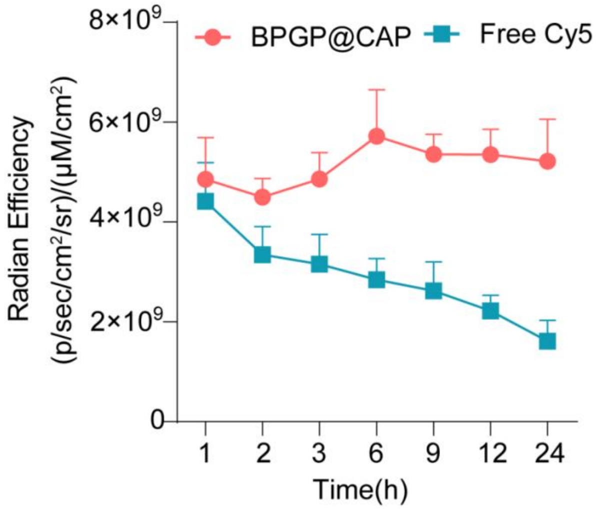

Figure S19. Total radiant efficiencies of tumors in the MFC tumor-bearing mice after treatment with free Cy5 and Cy5-labelled BPGP@CAP at different time points.

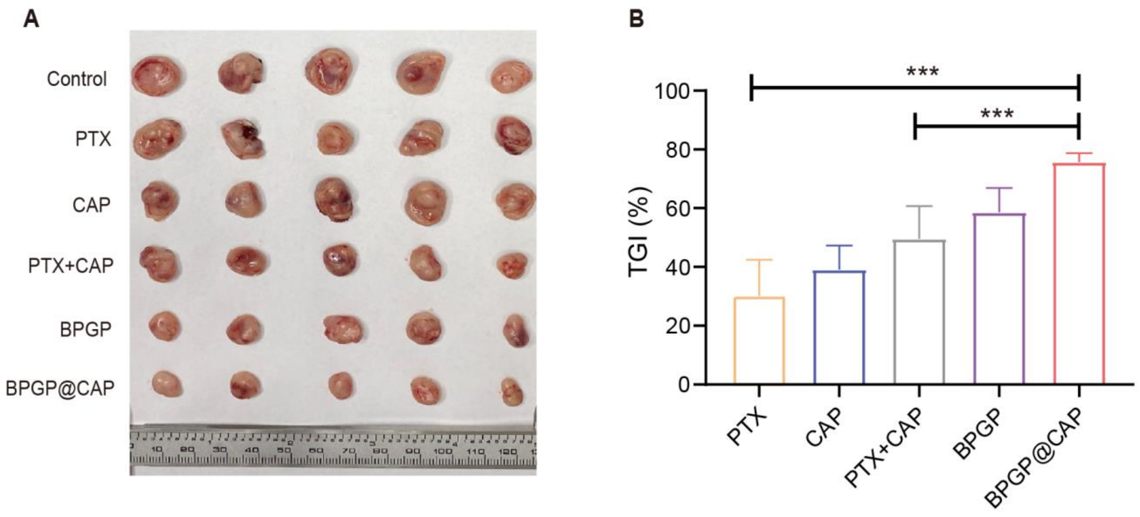

Figure S20. Photographs of tumors harvested from the mice at the end of the treatment schedule (A) and tumor growth inhibition (TGI) values in different treatment groups (B) (n = 5). Data were shown as mean ± SD. Statistical significance was determined using a two-sided unpaired Student's t-test, ****p* < 0.001.

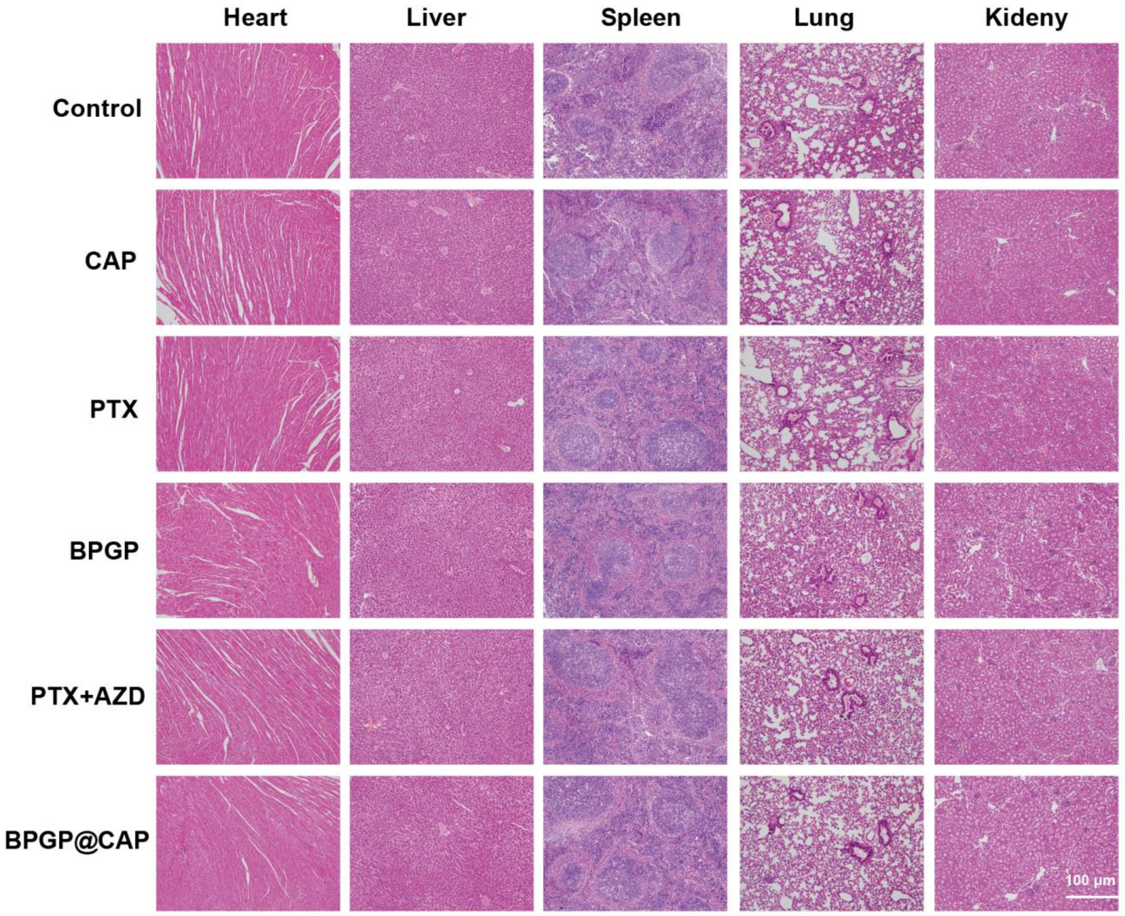

Figure S21. H&E staining of main organs from the mice after completion of the treatment with saline, PTX, CAP, PTX + CAP, BPGP, and BPGP@CAP. Scale bar = 100 μm.

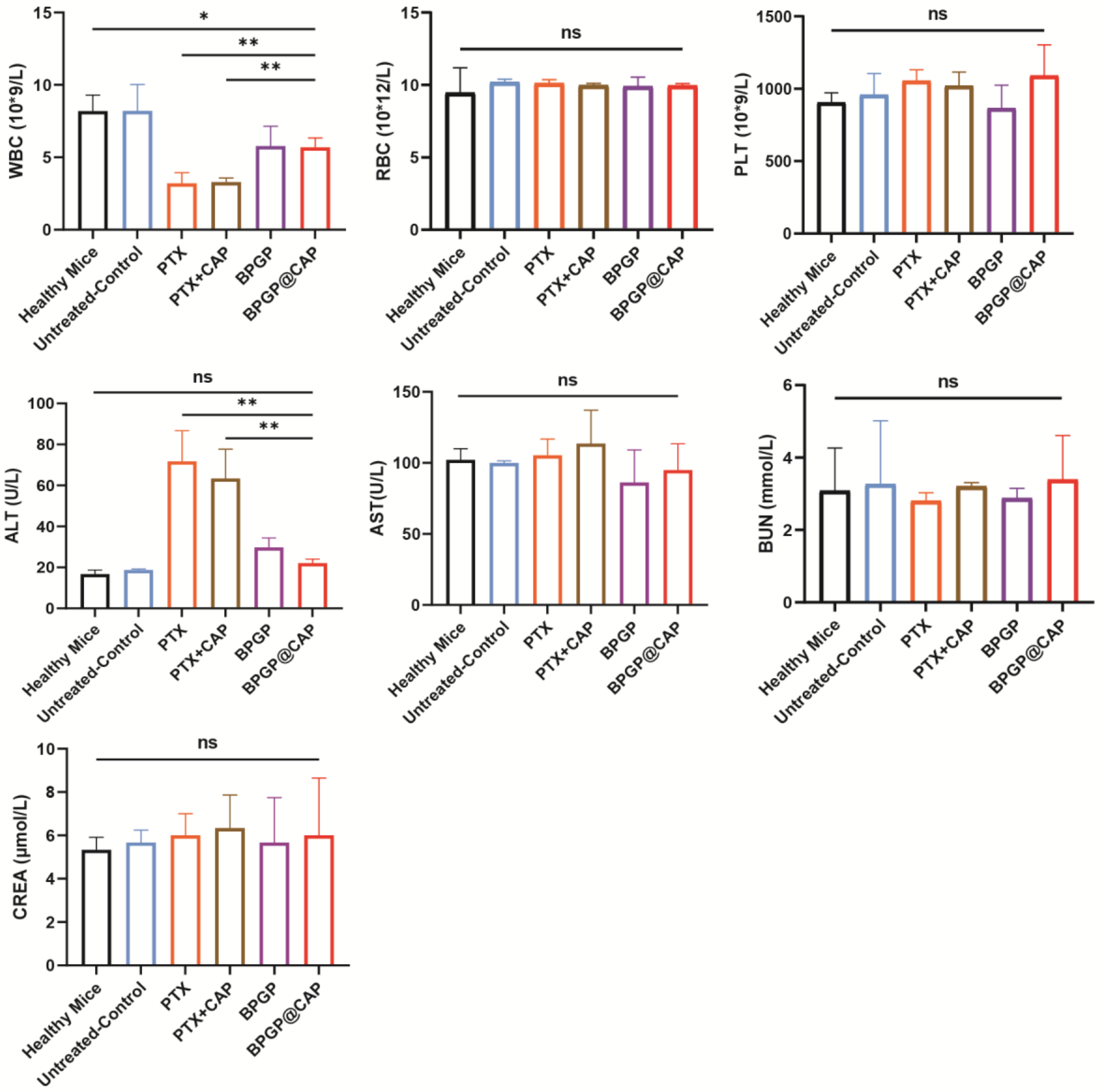

Figure S22. In vivo biosafety assessment of healthy mice and tumor-bearing mice after treatment with saline, PTX, CAP, PTX + CAP, BPGP and BPGP@CAP (n = 3). Routine blood analysis including white blood cells (WBC), red blood cells (RBC) and platelets (PLT); blood biochemistry tests including alanine aminotransferase (ALT), aspartate aminotransferase (AST), blood urea nitrogen (BUN) and creatinine (CREA) were performed.Data were shown as mean ± SD. Statistical significance was determined using a two-sided unpaired Student's *t*-test, $*p < 0.01$, $**p < 0.01$, $*** p < 0.001$ and ns for $p > 0.05$.

Table S1. The relationship between AKT expression and neoadjuvant chemotherapy response in gastric cancer

Factor	AKT-high (%)	AKT-low (%)	*P* value
TGR 0+1	4 (26.7)	7 (70.0)	0.032
TGR 2+3	11 (73.3)	3 (30.0)	

TRG, tumor regression grading

Table S2. Pharmacokinetic parameters for mice treated with BPGP@CAP and free Cy5 by fitting the data to a non-compartment model by PKSolver 2.0 software.

Parameter	Cy5	BPGP@CAP
AUC_{0-t}(ug/mL.min)	335.73±41.37	1126.61±136.90
$T_{1/2}$ (min)	160.85±33.59	488.49±61.74
C_{max} (ug/mL)	14.38±0.11	14.46±0.35
MRT (min)	129.4±5.03	245.17±12.40

Table S3. Scoring criteria for IHC

Positive cells	Scores	Staining intensity	Scores
<5%	0	no color	0
5%-25%	1	yellow	1
25-50%	2	light brown	2
50-75%	3	brown	3
>75%	4		

References:

[1] Z. Deng, S. Li, X. Jiang, R. Narain, *Macromolecules* **2009**, *42*, 6393.

[2] K. N. Plunkett, K. L. Berkowski, J. S. Moore, *Biomacromolecules* **2005**, *6*, 632.

[3] W. Wang, X. Zhang, Z. Li, D. Pan, H. Zhu, Z. Gu, J. Chen, H. Zhang, Q. Gong, K. Luo, *Carbohyd. Polym.* **2021**, *267, 118160.*

[4] Z. Duan, Y. Zhang, H. Zhu, L. Sun, H. Cai, B. Li, Q. Gong, Z. Gu, K. Luo, *ACS Appl. Mater. Inter.* **2017**, *9*, 3474.

[5] Y. Ma, L. Ma, Q. Guo, S. Zhang, *J. Exp. Clin. Cancer Res.* **2010**, *29, 85*.

[6] R. L. Camp, M. Dolled-Filhart, D. L. Rimm, *Clin. Cancer Res.* **2004**, *10*, 7252.

[7] F. Meng, Y. Yang, X. Wang, F. Cai, H. Liang, R. Zhang, J. Deng, *Surgery.* **2023**, S0039-6060(23)00430-0.
